# Oncogenic SRSF3 in health and diseases

**DOI:** 10.7150/ijbs.83368

**Published:** 2023-06-12

**Authors:** Rong Jia, Zhi-Ming Zheng

**Affiliations:** 1 The State Key Laboratory Breeding Base of Basic Science of Stomatology & Key Laboratory of Oral Biomedicine Ministry of Education, School & Hospital of Stomatology, Wuhan University, Wuhan, Hubei, China.; 2 Tumor Virus RNA Biology Section, HIV Dynamics and Replication Program, Center for Cancer Research, National Cancer Institute, National Institutes of Health, Frederick, Maryland, USA.

**Keywords:** SRSF3, Alternative splicing, Cancer, Diseases

## Abstract

Serine/arginine rich splicing factor 3 (SRSF3) is an important multi-functional splicing factor, and has attracted increasing attentions in the past thirty years. The importance of SRSF3 is evidenced by its impressively conserved protein sequences in all animals and alternative exon 4 which represents an autoregulatory mechanism to maintain its proper cellular expression level. New functions of SRSF3 have been continuously discovered recently, especially its oncogenic function. SRSF3 plays essential roles in many cellular processes by regulating almost all aspects of RNA biogenesis and processing of many target genes, and thus, contributes to tumorigenesis when overexpressed or disregulated. This review updates and highlights the gene, mRNA, and protein structure of SRSF3, the regulatory mechanisms of SRSF3 expression, and the characteristics of SRSF3 targets and binding sequences that contribute to SRSF3's diverse molecular and cellular functions in tumorigenesis and human diseases.

## 1. Introduction

The discovery of SRSF3 protein, also called SRp20 or SFRS3, goes back to a study of SR (serine/arginine rich) proteins with a mouse monoclonal anti-SR protein antibody, Mab104 in 1991 1, which recognizes all phosphorylated SR proteins in calf thymus 2. Subsequent sequence analysis showed that human *SRSF3* gene is the same as mouse *Srsf3* gene, which was previously called X16 3. SRSF3 is the smallest member of the splicing factor SR protein family which contains one or two N-terminal RNA recognition motifs (RRM) and a C-terminal serine and arginine-rich (RS) domain with consecutive RS repeats 4.

SRSF3, like other members of SR protein family, is considered as a key splicing factor in regulating constitutive and alternative pre-mRNA splicing and possibly RNA polyadenylation 5, 6. SRSF3 associates with the active sites of RNA polymerase II (pol II) in the nucleus 7 and interacts with the C-terminal domain of pol II 8, thus, promoting coupling of transcription and RNA splicing. Along with application of Cre-loxP knockout technology, Jumaa et al. in 1999 found that *SRSF3* is absolutely required for the development of embryos and the mice with *Srsf3* knockout are often embryonic lethal 9. Since then, more critical roles of SRSF3 have been revealed, including its roles in the development of human diseases and cancers. SRSF3 facilitates RNA export 10 by interacting with mRNA export receptor TAP/NXF1 11 and is tumorigenic as a proto-oncogene when overexpressed 12. SRSF3 promotes miRNA biogenesis by interacting and recruiting DROSHA to the Microprocessor cleavage site 13, 14. Recent genome-wide approaches have identified numerous RNA targets of SRSF3 in a mouse tumor cell line 15 and a human osteosarcoma cell line 16. This review will update what we have known about *SRSF3* gene structure and expression, SRSF3 protein structure and functions, and most importantly its critical roles in tumorigenesis.

## 2. Characteristics of SRSF3 gene, mRNA, and protein

### 2.1 SRSF3 gene and mRNA

**Figure 1:**
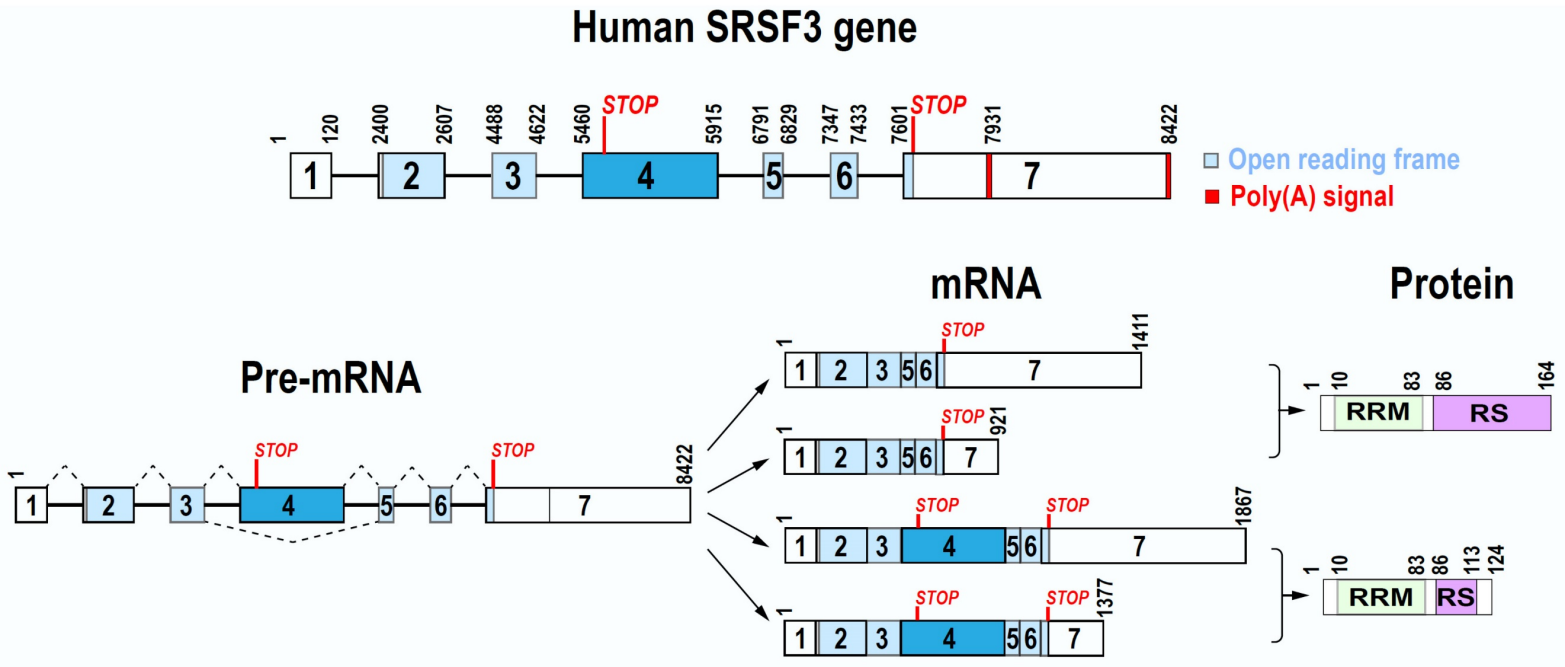
Gene, mRNA, and protein structures of human SRSF3. Human *SRSF3* gene contains seven exons and six introns, with exon 7 bearing two alternative poly(A) signals. Exon 4 is an alternative exon with a pre-mature stop codon. Because of alternative splicing and polyadenylation, *SRSF3* gene expresses four mRNA isoforms. Full-length SRSF3 protein (164 amino acid residues) is encoded by transcripts without exon 4, whereas the transcripts containing the exon 4 encode a truncated SRSF3 protein (124 amino acid residues).

*SRSF3* gene on human chr6p21.31 spans a 8422-bp region. Mouse *Srsf3* gene on chr17 is 9423-bp long. Both contain seven exons and six introns, of which the exon 4 bearing a stop codon is an alternative exon (Figure 1). Both promoter transcription start sites and polyadenylation signals of human *SRSF3* and mouse *Srsf3* genes are now well defined 17. We found that human *SRSF3* promoter contains a TATA box at -26 bp and mouse *Srsf3* promoter contains a TATA box at -27 bp upstream of each mapped transcription start site 17. Further upstream of the TATA box are multiple consensus transcription factor binding sites including AP1, CREB, SP1, and E2F 18. We discovered that both human *SRSF3* gene and mouse *Srsf3* gene have two polyadenylation signals (PAS), a major distal PAS “AUUAAA” and a minor proximal PAS “AAUAAA”. Because of exon 4 inclusion or exclusion and alternative usage of a distal or proximal PAS in pre-mRNA processing, human *SRSF3* or mouse *Srsf3* each expresses four different mRNA isoforms, of which the most abundant *SRSF3* or *Srsf3* mRNA is its isoform-1 mRNA less than ~1.5 kb in size 17.

The exon 4 in 456-nt size of both human *SRSF3* and mouse *Srsf3* is an alternative exon and contains a pre-mature stop codon 18, 19. Thus, it is normally excluded from a mature *SRSF3* mRNA during *SRSF3* pre-mRNA splicing. Interestingly, the nucleotide sequence of exon 4 is highly conserved from human to mouse and chimpanzee, with 100% identity, but only 1-2 nt mismatches to most mammals, more than 97% to birds (*Calypter anna* et al.), and 91.92% to fish (*Latimeria chalumnae*) (Figure S1), suggesting that the exon 4 in all animals is excluded during *SRSF3* pre-mRNA splicing. The *SRSF3* long isoform with exon 4 inclusion is mainly degraded by non-sense mediated decay (NMD) 20, 21 and thus, only a fraction encodes a truncated SRSF3 protein if survived from NMD. In contrast, the exon 4-skipped *SRSF3* mRNA is the major isoform of the RNA and has an open reading frame of 495 nt to encode a full-length SRSF3 protein (Figure 1). SRSF3 protein is well conserved among different animal species during evolution.

### 2.2 SRSF3 protein

#### 2.2.1 The structure of SRSF3 protein

Full-length SRSF3 protein contains 164 amino acid (aa) residues and bears a N-terminal RNA recognition motif (RRM) and a C-terminal arginine/serine-rich (RS) domain (Figure 1). Like other SR protein family members, the RRM domain of SRSF3 provides RNA-binding specificity, while the RS domain is involved in protein-protein interactions. The RRM (1-86 aa residues) of SRSF3 (SRp20) has a βαββαβ topology in a structure very similar to SRSF7 (9G8). Solution structure of the SRSF3 RRM and of the RRM in complex with the RNA sequence CAUC showed all 4 nts in contacting with SRSF3 RRM in a semi-sequence-specific manner with only the 5' cytosine recognized specifically by the SRSF3 RRM 22. A short arginine-rich peptide (aa 84-90, EKRSRNR) adjacent to the SRSF3 RRM, which does not contact RNA, is essential for interaction with an export factor Tip-associated protein TAP (NXF1) 22 to mediate RNA export 11.

As stated above, the *SRSF3* exon 4 contains a stop codon, inclusion of this exon 4 in *SRSF3* mRNA leads to encode a truncated, non-functional SRSF3 protein containing only a small part of the RS domain 18. Serum starving or oxidative stress induces exon 4 inclusion during *SRSF3* pre-mRNA splicing (See description below) 18, 23.

#### 2.2.2 Conservation of SRSF3 protein

SRSF3 protein is well conserved almost in all species of animal kingdom (Figure S2). By analyzing the conservation of SRSF3 protein sequences, impressively, we found that SRSF3 protein sequences are exactly the same in most mammals, including human, chimpanzee, monkey, dog, pig, fox, sheep, horse, cat, mouse, rat, beaver, and blue whale, except the hamster which has one aa substitution in the position of aa 28. Moreover, the birds, including sparrow, owl, chicken, and swan, share exactly the same SRSF3 protein sequences as human. As to reptilia, turtle also has the same SRSF3 protein sequence as human, while huge komodo dragon and small python all have only one substitution in aa 162, which is almost at the end of SRSF3 protein. Surprisingly, amphibian, exampled by caecilian or toad here, also have almost the same SRSF3 protein sequence as human with only a few aa substitutions, of which are mainly localized at the C-terminus.

#### 2.2.3 SRSF3 protein turnover and stability

SRSF3 protein stability is regulated by ubiquitination or neddylation. SRSF3 has at least two ubiquitinated sites, Lys23 and Lys85 24 and the ubiquitinated SRSF3 can be recognized by a monoclonal antibody against di-glycine-containing isopeptides. Under stress condition, SRSF3 can be neddylated at Lys11 and degraded by proteasome 25. However, the neddylation in SRSF3 Lys85, which is also responsible for stress granule assembly 26, did not affect SRSF3 protein stability 25.

#### 2.2.4 Phosphorylation of SRSF3 protein

In its steady state, SRSF3 protein is hypophosphorylated. Like other SR proteins, only under hypophosphorylated state, SRSF3 can bind NXF1 and promote RNA export 27, 28. However, phosphorylation status of SRSF3 is important for its functions and cellular localization. Phosphorylation is required for SRSF3 nuclear import and participating RNA splicing. SRSF3 protein are phosphorylated at around 11-12 serine residues in its RS domain by serine-arginine protein kinase 2 (SRPK2). The SRSF3 RS domain also contains the Akt consensus site (RxRxxS/T) 29 and thus can be phosphorylated by the PI3K-Akt signal pathway 30. Phosphatase 1 catalytic subunit α (PP1α) dephosphorylates SRSF3 31. SRSF1 (SF2/ASF) has two RRM domains, RRM1 and RRM2. Phosphoryl content of the SRSF1 RS domain could be protected by the RRM2 domain from dephosphorylation by phosphatases. SRSF3 lacks the extra RRM domain, and its phosphoryl content is more sensitive to phosphatases than SRSF1, suggesting a relative rapid dynamic switch of its phosphorylation status in correlation with the dynamic nature of spliceosome 31. SRSF3 protein can be also dephosphorylated by PPM1G (also named PP2Cγ), a serine/threonine protein phosphatase, in hepatocellular carcinoma cells 32, although PPM1G-mediated dephosphorylation is involved in formation of the spliceosome 33.

#### 2.2.5 Shuttle of SRSF3 between the nucleus and cytoplasm

SRSF3 protein mainly stays in the nucleus revealed by immunohistochemistry, immunocytochemistry, and nuclear and cytoplasmic fractionation analyses (exampled by ref. 34, 35). However, similar to SRSF1 and SRSF7, SRSF3 does shuttle between the nucleus and cytoplasm, which facilitates mRNA export and stability in the cytoplasm. The phosphorylated RS domain and stable RNA binding mediated by the RRM domain of SRSF3 are required for shuttling 36. Phosphorylated RS domain is recognized and imported into the nucleus by transportin-SR2 (TRN-SR2) 37. SRSF3 RS domain contains not only a nuclear localization signal, but also directs itself to nuclear speckles 38.

Under certain conditions, SRSF3 may distribute to the cytoplasm. Viruses can promote cytoplasmic localization of SRSF3 to meet their requirements. For example, poliovirus 2A proteinase and human rhinovirus 16 2A proteinase can induce cytoplasmic relocalization of SRSF3 possibly by their cleavage of nuclear pore complex proteins 39. Under osmotic stress condition, SRSF3 interacts with activated p38α MAPK in cardiomyocytes, resulting in partial SRSF3 distribution in the cytoplasm 40.

#### 2.2.6 SRSF3 binds to chromatin

When SRSF3 stays in the nucleus, SRSF3 mainly participates RNA splicing in active sites of RNA polymerase II transcription 7. In addition, it also binds to chromatin and both SRSF3 and SRSF1 are associated with interphase chromatin through histone H3. When histone H3 serine 10 is phosphorylated during mitosis, SRSF3 and SRSF1 are disassociated with hyperphosphorylated mitotic chromosomes, but re-associated with chromatin in the late M-phase. Phosphorylation of the RS domain of SRSF3 is required for its association with chromatin 41. We found that SRSF3 knockdown induced cell cycle G2/M arrest 12, which may be partially related to its binding to chromatin. However, the exact roles of SRSF3 in association with chromatin remains to be determined.

DNA methylation affects the splicing of ~20% genomic alternative exons 42. High DNA methylation in the gene body regions are associated with increased inclusion of alternatively spliced exons 43. Histone H3 plays central roles in the association of chromatin with spliceosome as well SRSF3. Heterochromatin protein 1 (HP1) binds H3K9me3 in the chromatin and recruit SRSF3, which is transferred to the pre-mRNA and regulates alternative splicing 42. H3K9 demethylase KDM3A is phosphorylated by protein kinase A (PKA) and recognizes H3K9 methylation to recruit SRSF3 to its target pre-mRNA, however, independent of its demethylase activity 44.

### 2.3 Homology between SRSF3 and other SR family members

So far, at least 12 SR proteins, SRSF1 to SRSF12 (SRrp35), have been discovered. All SR proteins have one or two N-terminal RRM domains and one C-terminal RS domain. Even though it is the smallest SR protein, SRSF3 can functionally complement splicing-deficient cytoplasmic S100 extracts 5, 45. SRSF3 RS domain contains 14 “RS” or “SR” dipeptide repeats, which is similar to other members. However, the RRM domain of SRSF3 is significantly different from most of other members, which may suggest the RRM specificity for SRSF3. A few residues are conserved across SR family, such as Phe30, Gly34, Val51, Phe53, Asp55, Ala59, and Ala62. The sequence of “FAFVEFEDPRDAADA” in the middle of RRM domain is conserved in most members, except SRSF11 (p54). Interestingly, the RRM domain of SRSF3 is around 75% identity to that of SRSF7, suggesting that SRSF3 may recognize similar RNA motifs as SRSF7 (Figure S3).

## 3. SRSF3 expression and regulatory mechanisms

According to the data of human protein atlas (HPA) project, *SRSF3* transcripts could be detected in almost all 27 different normal tissues, even though the expression levels are quite low in pancreas and salivary gland tissues 46 as showing in the webpage of human *SRSF3* gene in Genbank. In mouse, the expression levels of SRSF3 is also significantly different in various tissues. Thymus, testis and spleen have high SRSF3 expression, while heart, liver and kidney have low or undetectable SRSF3 3.

**Figure 2:**
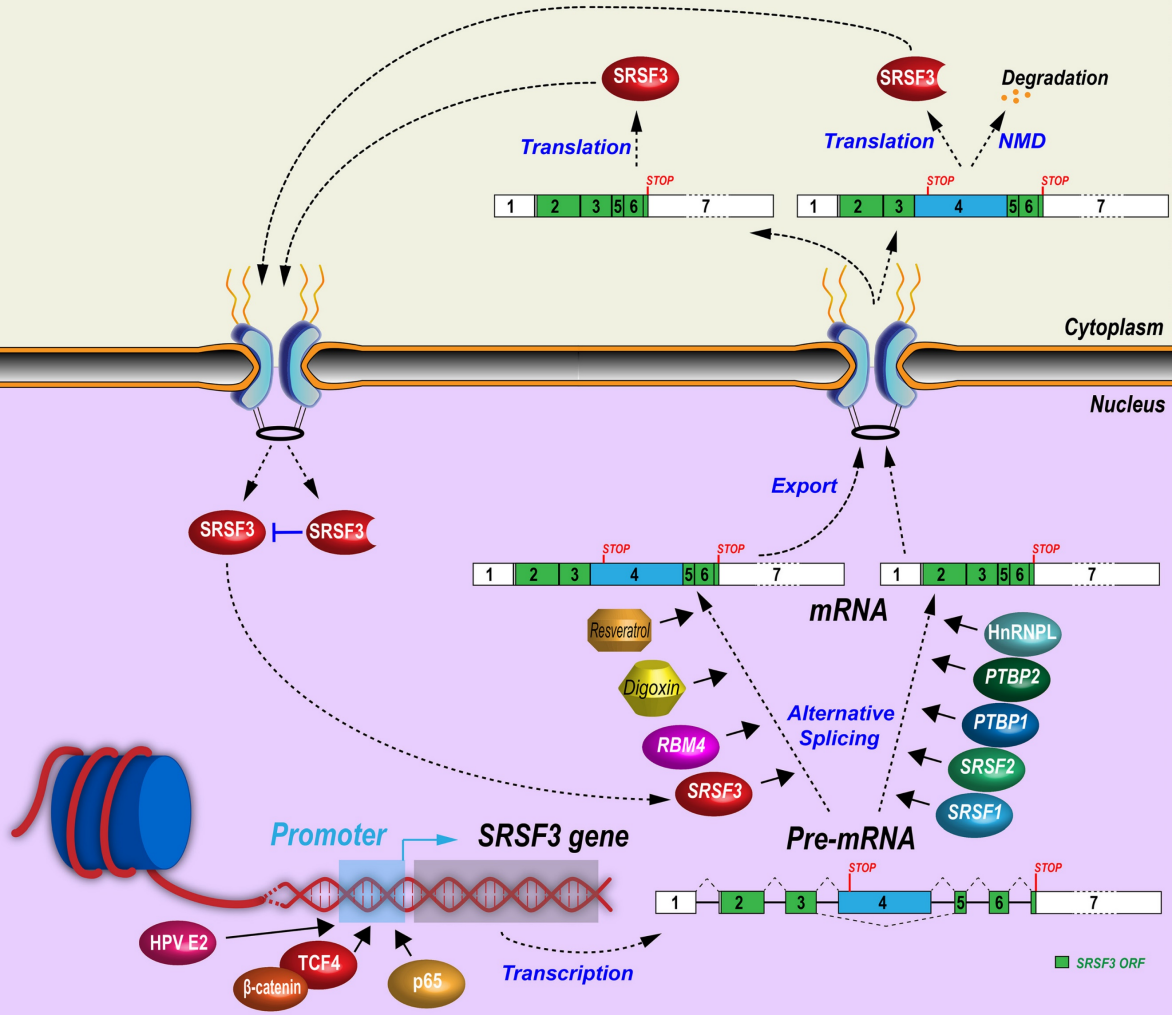
Regulatory mechanisms of SRSF3 expression. Expression of SRSF3 is regulated at the transcription level by transcription factors or at the post-transcriptional levels by alternative splicing of exon 4.

SRSF3 expression is absolutely required for embryonic development. Knockout of *Srsf3* in mice blocked embryonic development before the blastocyst stage 9. Conditional *Srsf3* knockout in cardiomyocytes is lethel during embryonic development 47 and in neural crest cells affects mouse facial processes 30 SRSF3 expression has been reported in many types of cells, and is essential for cell proliferation 12, 48 However, overexpression of SRSF3 is tumorigenesis 12 Therefore, SRSF3 expression is regulated sophisticatedly and has to be maintained in balance in different cell types and at different stage of cell cycles (Figure 2).

### 3.1 *SRSF3* promoter and its transcription regulation

Although *SRSF3* promoter and its enhancers/silencers have not been well studied, several transcription factors or co-factors have been demonstrated to enhance SRSF3 gene transcription through its promoter. β-catenin is often mutated and escapes from degradation in tumors. Increased β-catenin translocates into the nucleus and interacts with TCF/LEF transcription factor family members to promote target gene expression and tumorigenesis 49. A constitutively active mutant β-catenin can significantly increase SRSF3 expression by activating its transcription through the binding of TCF4 to an undefined region between nucleotides -225 and -1282 of *SRSF3* promoter 50. β-catenin is a central mediator of Wnt signaling pathway and CD133^+^ (a stemness marker for cancer stem cells) colon cancer stem cells express more SRSF3 than CD133^-^ non-stem cancer cells. Activation of Wnt signaling pathway by Wnt3a protein significantly increases SRSF3 expression in colon cancer cells 51.

Other signal pathways related to transcriptional regulation have been explored. TGF-β1 enhances phosphorylation of p38 MAPK, which recruits NF-κB p65 binding to the *SRSF3* promoter to increase SRSF3 expression 52. Hepatocyte growth factor induces AKT phosphorylation to downregulate SRSF3 53. Insulin induces the levels of all phosphorylated SR proteins, including SRSF3, in rat hepatocytes 54, but promotes SRSF3 protein degradation by proteasome in human myeloblastic HL-60 cells 55. Consistently, insulin-like growth factor 2 (IGF2) activates AKT (pSer473) and ERK1/2 (pThr202/Tyr204) phosphorylation to reduce SRSF3 expression in HepG2 cells. The IGF2 effect was specific for SRSF3 as other SR proteins did not change in expression 56. Phospholipase C-β1 (PLC-β1) is a key nucleic enzyme for cell proliferation and differentiation by promotion of G1 cell cycle progression 57. PLC-β1 interacts with SRSF3 directly in the nucleus to decrease SRSF3 protein level in Friend erythroleukemia cells 58, whereas hypoxia induces SRSF3 expression in human head and neck squamous cells 59. HPV16 infection increases SRSF3 expression in undifferentiated keratinocytes through E2 protein 60 binding to *SRSF3* promoter 61, but the increased *SRSF3* promotes RNA splicing of viral early transcripts to disrupt integrity of the E2 ORF and therefore repress E2 expression, providing a negative feedback control of SRSF3 expression 62.

### 3.2 Alternative splicing of poison exon 4

Alternative splicing of the exon 4 bearing a stop codon is a very efficient way in regulating SRSF3 expression. Similar to SRSF3 alternative exon 4, most of other SR family members contain an alternative “poison” exon to negatively regulate their expression 19. *SRSF3* mRNAs with exon 4 inclusion encode a truncated SRSF3 protein. Only *SRSF3* mRNAs with exon 4 exclusion can encode a full-length SRSF3 protein. Therefore, the increasing inclusion of exon 4 in *SRSF3* RNA splicing will reduce the expression of full-length SRSF3 protein.

A number of mechanisms controlling *SRSF3* exon 4 splicing have been revealed. First, SRSF3 itself can promote exon 4 inclusion and form a negative feedback regulation. Overexpression of Srsf3 significantly increases its own exon 4 inclusion in K46 cells (a murine B-cell lymphoma) 63. Second, many other splicing factors regulate exon 4 splicing. In K46 cells, oncogenic SRSF1 and SRSF2 (SC35) suppress exon 4 inclusion and lead to the increase of SRSF3 protein 63, 64, but in U2OS cells, SRSF1 promotes inclusion of *SRSF3* exon 4 16. A circRNA derived from SMARCA5 gene, cicrSMARCA5 which is not characterized carefully with unknown copy number and cellular locations, appears to increase the inclusion of *SRSF3* exon 4 in glioblastoma cells 65. Oncogenic splicing factors PTBP1 and PTBP2 suppress exon 4 inclusion *via* an exonic splicing suppressor, “UUUCU” 21. In contrast, tumor suppressor RBM4 66 reverses PTBP1-mediated skipping of exon 4 inclusion *via* an intronic CU-element 67. In hepatocytes and in PLC/PRF/5 and H358 cells, splicing factor SLU7 inhibits inclusion of SRSF3 exon 4 68, 69. HnRNP L promotes SRSF3 full-length protein expression also by inhibiting exon 4 inclusion in oral squamous cell carcinoma cells 70. Third, exon 4 splicing is associated with cell proliferation and stress. Mouse *Srsf3* isoform with a poison exon 4 inclusion was high in starved K46 cells, and quickly decreased after serum stimulation 18. Oxidative stress-induced by arsenite inhibits NMD to promote stability of *SRSF3* isoform RNA with exon 4 inclusion in a human colon cancer cell line, HCT16 cells 23.

Other chemicals/drugs regulating exon 4 splicing have been reported. Digoxin is a cardiac glycoside drug used in the treatment of heart failure and arrhythmia. Digoxin represses SRSF3 expression by promoting *SRSF3* exon 4 inclusion in a dose- and time-dependent manner 71. Resveratrol is a small biocidal molecule widely produced, upon stress, by plants, including grape and peanut. Resveratrol and its derivatives show promising therapeutic effects in cancer, cardiovascular diseases, neurodegenerative diseases, infections, and so on 72. Interestingly, resveratrol treatment of HEK293 cells increased SRSF3 exon 4 inclusion in a dose-dependent manner 73.

## 4. Function and molecular targets of SRSF3

### 4.1 Alternative RNA splicing targets of SRSF3 (Table 1)

SRSF3 interacts with RNA motifs to regulate RNA alternative splicing. SRSF3 also interacts with the C-terminal domain (CTD) of RNA polymerase II to regulate transcription-coupled alternative RNA splicing. Both SRSF1 and SRSF3 regulate the inclusion of an alternative exon of fibronectin gene, but deletion of the Pol II CTD was found only affecting the regulatory function of SRSF3, not SRSF1 8.

#### 4.1.1 High throughput analysis of SRSF3 targets and binding motifs

As an important splicing factor, SRSF3 regulates the alternative RNA splicing of hundreds of target genes. High-throughput methods were used to identify SRSF3 targets and binding motifs. We performed a genome-wide analysis of SRSF3 target genes and alternative RNA splicing events by using a human splicearray platform in human osteosarcoma U2OS cells with versus without SRSF3 knockdown 16. We showed that SRSF3 regulates the transcription of 60 genes, including *ERRFI1* 74, *ANXA1* 75 and *TGFB2* 76, and 182 RNA splicing events in 164 genes, including *EP300*, *PUS3*, *CLINT1*, *PKP4*, *KIF23*, *CHK1*, *SMC2*, *CKLF*, *MAP4*, *MBNL1*, *MELK*, *DDX5*, *PABPC1*, *MAP4K4*, *SP1* and *SRSF1* 16. Many SRSF3 target genes and their alternative RNA splicing events are significantly associated with tumorigenesis, such as *EP300* 77, mitogen-activated protein 4 kinase 4 (*MAP4K4*) 78, and *SRSF1* 79. Motif analysis showed that two SRSF3-binding motifs, CCAGC(G)C and A(G)CAGCA, are enriched to the RNA alternative exons. For example, SRSF3 promotes EP300 exon 14 inclusion through motif CCAGCC 16. Later, we performed another genome-wide analysis of SRSF3 target genes and alternative splicing events by using a mouse splicearray platform in mouse fibroblast MEF3T3 cells with SRSF3 overexpression. The top lists also showed that SRSF3 regulated the expression of 11 genes and 139 splicing events in 134 genes 80. Surprisingly, among these targets, alternative RNA splicing events in expression of *ILF3* (interleukin enhancer binding factor 3) gene are the only conserved target splicing events of SRSF3 from human U2OS cells to mouse MEF3T3 cells.

RNA-seq has been widely used to analyze the effects of *Srsf3* depletion on alternative splicing of mouse neonatal cardiomyocytes 47, neural crest cells 30, and mouse oocytes 81, and the roles of SRSF3 in development of human glioblastoma 82, 83. Conditional knockout of *Srsf3* in mouse oocytes led to sterility. Single cell RNA-seq found a number of unannotated splicing events in mouse *Srsf3* knockout oocytes, the depression of some transposable elements, and some known alternative splicing events of the genes related to oocytes maturation, such as exon 11 skipping of *Brd8* and mutually exclusive of *Pdlim7* exon 5 and 6 81. High level of SRSF3 expression in glioblastoma has been noticed 80 and is essential for survival of glioblastoma cells 82, 83. SRSF3 in glioblastoma-derived GSC83 and 528 cells induces the up-regulation of oncogenic splicing variants of ETV1 and NDE1 82. Functional SRSF3 in glioblastoma cells U-87 MG and U-118 MG is related to PDGFRB-activated PI3K-AKT-ERK pathway 83. Oncogene HER2 produces multiple isoforms by alternative splicing. SRSF3 binds to exon 15 and inhibits the inclusion of downstream exons to increase the expression of oncogenic HER2 isoform 84.

Seeking for SRSF3-specific binding sites was initially undertaken by SELEX and identified a (A/U)C(A/U)(A/U)C motif being specific for both SRSF3 and SRSF7 85. Because of lacking high quality of anti-SRSF3 antibody for immunoprecipitation, various *in vivo* RNA-binding landscape studies of SRSF3 were undertaken by expression of a SRSF3-tagged fusion in the presence of endogenous SRSF3. Cautions should be taken in carrying out such a study because a SRSF3-tagged fusion may not truly capture the target RNAs of the endogenous SRSF3 and the excessive expression of SRSF3-tagged fusion may affect the overall gene expression profile from normal endogenous SRSF3 at a physiological or pathological condition. In mouse pluripotent embryonal carcinoma P19 cells with SRSF3-GFP expression, anti-GFP CLIP discovered hundreds of transcripts in association with SRSF3-GFP, including PTBP1, SLAIN2 and the intron-retained mRNA isoforms SRSF3 and SRSF7 15] 86, and identified many SRSF3-GFP binding motifs with a pentamer sequence UCAUC, CUUCA, CAUCA, CAUCU, or UCAAC 87. These pentamers are similar to the core motifs, CUCKUCY, identified by an *in vitro* SELEX screening assay 88. Further studies using mouse neonatal cardiomyocytes expressing SRSF3-GFP by anti-GFP CLIP and RNA-seq showed that SRSF3 binding sites identified are mainly in the exclusion-enhanced exons, but in the flanking introns or exons for the inclusion-enhanced exons 47. To more specifically identify targets and binding sites of SRSF3 in cells, a TRIBE (targets of RNA binding sites by editing) 89 technique by expression of SRSF3 fused with an-adenosine deaminase domain in human embryonic H9 cells was established in combination with RNA-seq analysis to identify SRSF3 target RNAs bearing A to G editing events. This technique identified 3888 confident binding sites with conserved CA-rich sequences in RNAs from 1222 genes 90. In mouse hepatocytes, Srsf3 deletion caused insulin receptor gene (Insr) exon 11 skipping and produced isoform INSR-A in *Srsf3* knockout hepatocytes, which can be strongly activated by IGF2 treatment and led to significantly enhance cell proliferation 91. In human *INSR* gene, exon 11 also contains an exonic splicing enhancer of CUCUUC interacting with SRSF3 92.

In addition to the varables derived from different assay platforms being used in the studies, the targets and binding motifs of SRSF3 identified may vary widely with different species and cell types, even though SRSF3 protein sequence is the same in both human and mouse.

#### 4.1.2 Other splicing factors as a SRSF3 target

SRSF3 regulates not only its own exon 4 splicing but also other splicing factors' alternative splicing events. For example, MBNL1 (muscle blind-like 1) is a splicing factor with 400-aa residues and has 10 exons. Alternative exons 4 and 7 included in full-length *MBNL1* is required for nuclear localization and dimerization 93. MBNL1 inhibits the inclusion of its own exon 7. However, SRSF3 breaks its autoregulatory mechanism and enhances the inclusion of exons 4 and 7 *via* exonic CA-rich elements 94 [16. Besides its own “poison” exon 4, SRSF3 also promotes the poison exons inclusion of *SRSF1* 16 and *SRSF7* 87 and shares many co-targets with TDP43 (TAR DNA-binding protein, also called TARDBP), an hnRNP family splicing factor 95. For example, SRSF3 and TDP43 inhibited inclusion of *SYNGAP1* exon 14 by binding to an exon 14 and an intron 15 motif, respectively, in a cooperative manner and knockdown of either SRSF3 or TDP43 expression promotes inclusion of the exon 14 95.

### 4.2 SRSF3 regulation of other RNA processing steps

**Figure 3:**
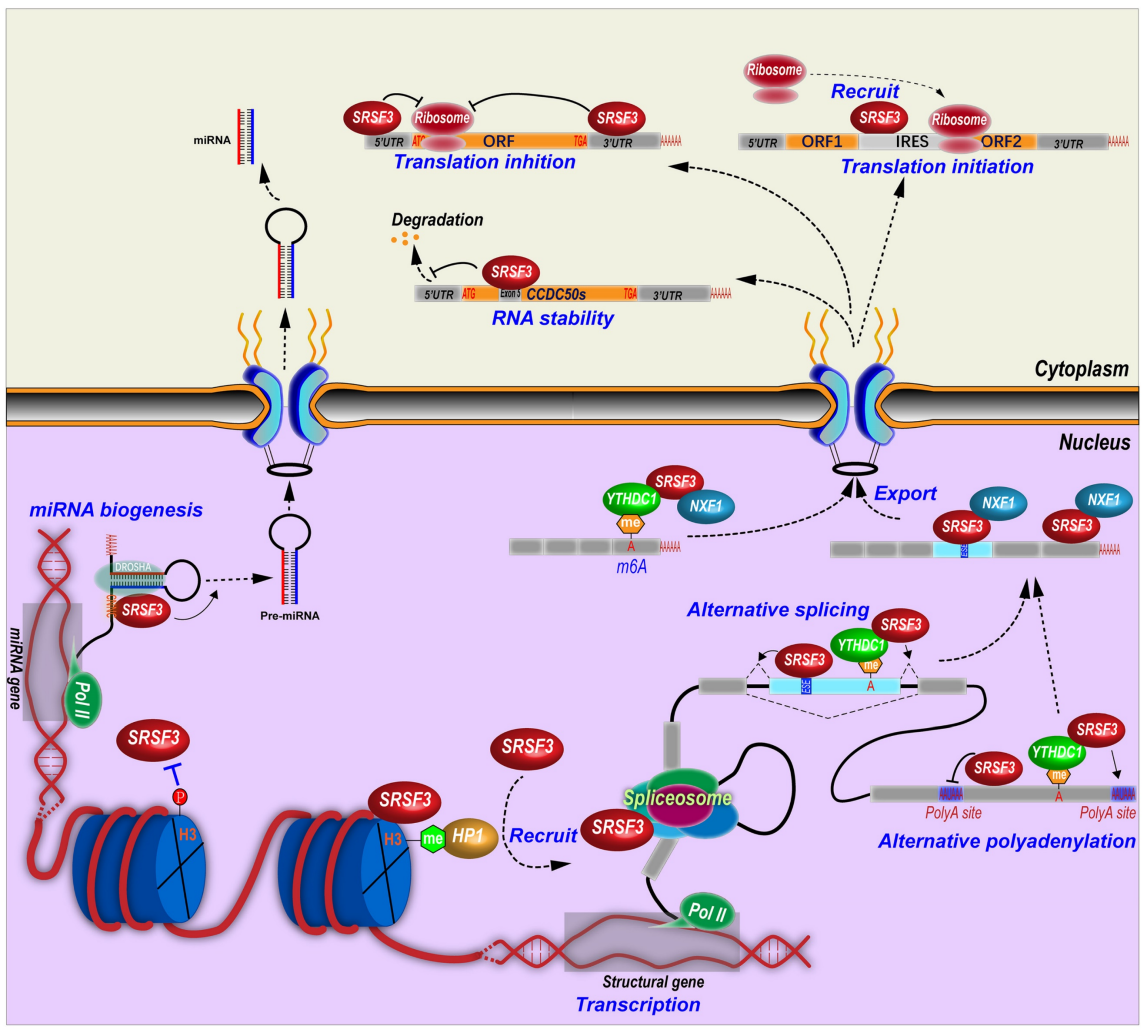
Molecular mechanisms of SRSF3 in regulation of RNA biogenesis and processing. SRSF3 regulates gene transcription, alternative splicing and polyadenylation of pre-mRNAs, RNA export, stability, and translation, and miRNA biogenesis.

Other than RNA splicing regulation, SRSF3 is involved in almost all aspects of RNA biogenesis and processing including gene transcription, RNA polyadenylation, export, stability, translation, and miRNA biogenesis (Figure 3).

#### 4.2.1 Termination of transcription

In *C. elegans*, depletion of *Srsf3* impaired transcription termination 96. The relative mechanism remains unclear. In mammalian cells, SRSF3 may also regulate transcriptional termination by interaction with RNA polyadenylation cleavage factor CFIm 97.

#### 4.2.2 SRSF3 and polyadenylation

Many genes contain alternative polyadenylation sites and produces transcripts with different length of 3' untranslated regions (3' UTRs), which affects RNA stability, translation efficiency, and so on 98. In fact, SRSF3 can regulate alternative polyadenylation in many genes. SRSF3 knockdown was found to globally promote the usage of proximal alternative polyadenylation and produce transcripts with shorten 3' UTRs, which in general facilitates protein translation, in human HEK293T cells 99. SRSF3 binds more frequently to proximal polyadenylation sites than distal sites, and suppresses the usage of proximal sites. Notably, many genes related to cell division and cell cycle switch utilize the proximal polyadenylation sites that could be upregulated upon SRSF3 knockdown, such as PTEN, which inhibits cell proliferation and induces senescence in 293T, NIH3T3, and human umbilical vein endothelial cells 99. Mouse *Srsf3* promotes the usage of distal polyadenylation sites in mouse pluripotent P19 cells by binding to the motifs around proximal polyadenylation sites. This is because *Srsf3* prevents the proximal site binding of *Srsf7*, which promotes the usage of proximal sites 100. In addition, SRSF3 also regulates the selection of polyadenylation sites *via* an indirect mechanism. The cleavage and polyadenylation (CPA) of pre-mRNA are mainly mediated by the CPA machines including cleavage factor Im (CFIm) complex, which inhibit proximal polyadenylation site usage 101. Mouse Srsf3 knockdown reduces Cpsf6 expression, a component of CFIm complex, due to the open reading framing shift caused by the inclusion of an alternative exon in intron 5 or the exclusion of exon 6 in *Cpsf6* transcripts 100. SRSF3 binds an intronic splicing enhancer to promote the inclusion of *CT/CGRP* gene exon 4 and the usage of an alternative polyadenylation site in exon 4 by interacting with cleavage stimulation factor CstF 6.

#### 4.2.3 SRSF3 promotes RNA export

SRSF3 interacts with a 22-nt motif (ACAACAAGAAGACGCGCAUCAU) in mouse histone H2a RNA to facilitate the intronless H2a RNA export 10 and binds other histone RNAs 87. SRSF3 interacts with the N-terminus of NXF1, the major export receptor of mRNAs 11. SRSF3 promotes the recruitment of NXF1 to last exons of mRNAs and forms an RNA-SRSF3-NXF1 complex to be exported to the cytoplasm. Thus, SRSF3 determines the sequence specificity of exported target mRNAs 28. This interaction between SRSF3 and NXF1 is mediated by an arginine-rich peptide near the RRM domain of SRSF3 22.

*PDCD4* (Programmed cell death 4) has a novel exon in intron 2. SRSF3 knockdown promotes the nuclear export of the transcripts with this novel exon, but has no effects on the transcripts without this novel exon 102. *PD-1* (Programmed cell death-1) mRNA nuclear export is also promoted by the interaction between SRSF3 and NXF1 52.

NANOG is a core pluripotency transcription factor. SRSF3 facilitates mouse Nanog mRNA export and protein expression indirectly through regulation of *Nxf1* RNA splicing. SRSF3 promotes *Nxf1* intron 10 retention and increases a short isoform of Nxf1 protein which lacks interaction with the nuclear pore complex 103, suggesting the presence of a potential feedback regulatory mechanism by which cells can maintain relative stable level of NXF1 and RNA export levels upon SRSF3 expression.

#### 4.2.4 SRSF3 regulates RNA stability

In the nucleus, SRSF3 plays different roles in RNA stability. SRSF3 controls the expression of the core components of NEXT (nuclear exosome targeting) complex, including RBM7, ZCCHC8 and hMTR4 104, indicating SRSF3 is linked to a nuclear RNA decay pathway.

Mouse Srsf3 depletion increases mTOR intron 5 retention, which leads to open reading frame shift and the loss of mTOR enzyme activity. Reduced expression of mTOR full-length protein cannot maintain eIF4E-BP1 phosphorylation, and thus impairs eIF4E-BP1 binding to eIF4E, resulting in disassociation of eIF4E from the mRNA cap. Finally, mRNA decapping of contraction-related genes leads to their RNA degradation in adult mouse heart with Srsf3 knockout 47.

#### 4.2.5 SRSF3 regulates RNA m6A modification

N6-methyladenosine (m6A) alters RNA alternative splicing. YTHDC1, a N6-methyladenosine (m6A) modified RNA binding protein or a m6A “reader”, displays similar regulatory splicing patterns as to SRSF3 but opposite to SRSF10 (SRrp40). YTHDC1 binds m6A modified exons and recruits SRSF3 to regulate exon alternative splicing, but blocks SRSF10 binding 105. YTHDC1 regulates alternative polyadenylation. SRSF3 interacts with YTHDC1 and identifies the m6A modified sites around last exon, and then promotes the usage of distal alternative polyadenylation sites and produces a long 3'UTR region 106. SRSF3 binds to m6A modification in *ANRIL* exon 1 and promotes the expression of *ANRIL* long isoform but inhibits the expression of *ANRIL* short isoform 107. Knockdown of SRSF3 leads to decrease m6A modification level of *ANRIL* in pancreatic cancer cells 107. SRSF3 facilitates the nuclear export of N6-methyladenosine (m6A) modified RNA. YTHDC1 is required for the nuclear export of m6A-containing RNA by interacting with hypo-phosphorylated SRSF3, which bridges YTHDC1 and the export receptor NXF1 108.

#### 4.2.6 SRSF3 in miRNA biogenesis and miRNA pathways

SRSF3 regulates the expression of miRNAs. By using miRNA microarray assays, we identified at least 20 miRNAs were regulated by SRSF3, including a subset of oncogenic or tumor suppressive miRNAs 16.

Mechanically, SRSF3 promotes miRNA biogenesis by binding to a CNNC (N represents any nucleotide) motif. This CNNC motif is around 17-18 nt downstream of the Drosha cleavage site and recruits Drosha to the basal junction and enhances the processing of the pri-miRNAs 13, 14. This CNNC motif is correlated with a CAUC sequence interacting with the SRSF3 RRM domain 22. More specifically, a single G to A substitution in mir-30c-1 gene facilitates SRSF3 binding and increases miR-30c-1 biogenesis and expression level in breast and gastric cancer patients 109. Deletion of a “CNNC” motif in miR-3131 gene interferes the binding of SRSF3 and the biogenesis of mature miR-3131 110.

SRSF3 also indirectly regulates microRNA expression. SRSF3 inhibits expression of miR-132-3p and miR-212-3p by increasing the expression of RE1-silencing transcription factor (REST), which is a transcription repressor 111. Expression of miR-1908 is transactivated by NF-κB. SRSF3 promotes nuclear translocation of p65 (RelA), a subunit of dimeric NF-kB, to increase the expression of miR-1908 112.

## 5. The roles of SRSF3 in cancer

In 2010, we discovered that SRSF3 is a proto-oncogene and overexpressed in numerous types of cancers and demonstrated that this overexpression of SRSF3 in cancer cells is critical for cancer cell proliferation and tumor induction and maintenance^12^. Following our observation, many other studies including ours have explored the expression, functions and regulatory mechanisms of SRSF3 in various cancers. Up to date, it becomes clear that SRSF3 is involved in the almost all aspects of carcinogenesis.

### 5.1 Expression of SRSF3 in cancers

SRSF3 overexpression is strongly positively associated with various cancers. and is overexpressed in almost all solid cancers examined, including cancers of the cervix, lung, breast, esophagus, stomach, skin, bladder, colon, liver, thyroid, and kidney, mesenchymal tissue-derived tumors, including rhadbomyosarcoma, hemangioendothelioma, hemangiopericytoma, neurofibroma, neurilemmoma, liposarcoma, leiomyosarcoma, histiocytoma, and synovial sarcoma, and B-cell lymphomas 12. Subsequently, other studies confirmed our initial findings of SRSF3 overexpression in breast cancer 113, colon cancer 114, or cervical cancer 115, colorectal cancer 67, 116, and other types of cancers including oral squamous cell carcinoma 117, ovarian cancer 118, 119, and glioblastoma 80, 82.

In contrast to most cancers, the role of SRSF3 in hepatocellular carcinoma is complicated. *Srsf3* knockout in mice appears to induce hepatocellular carcinoma 91 and overexpression of SRSF3 suppresses hepatocellular carcinoma cell proliferation. Ser/Thr phosphatase PPM1G promotes tumorigenesis of hepatocellular carcinoma cells by dephosphorylating SRSF3 32. However, *SRSF3* mRNA levels are upregulated in hepatocellular carcinoma tissues when compared with no-tumor tissues 120. While *SRSF3* mRNA is increased, the significant reduction of SRSF3 protein, not other SR proteins, being accompanied by induction of DNA damage in hepatocellular carcinoma, appears to be related to IGF2 overexpression and activation of AKT and ERK signaling 56. Notably, SRSF3 is essential to prevent cell apoptosis 12. More studies are required to uncover the exact roles and expression regulatory mechanisms of SRSF3 in hepatocellular carcinogenesis.

### 5.2 SRSF3 regulates cellular functions associated with tumorigenesis (Figure 4)

#### 5.2.1 Cancer cell proliferation and cell cycle progression

Malignant cells are characterized by accelerated proliferation and cell cycle progression. SRSF3 is a proto-oncogene and plays important roles in cell proliferation and cell cycle progression essential for tumorigenesis. SRSF3 overexpression promotes cell cycle progression by pushing the cells quickly through G2/M phase in synchronized mouse embryonic fibroblast cells 12. Consistently, SRSF3 knockdown induces cell cycle arrest in G2/M stage in U2OS and HeLa cancer cells 12. Mechanically, SRSF3 promotes expression of FOXM1, PLK1, and Cdc25B, three important cell cycle regulators 12. SRSF3 prevents the inclusion of *FOXM1* exon 9 for FOXM1a production, but enhances exon 9 exclusion to produce FOXM1b and FOXM1c, which are the key transcriptional factors for cell cycle G2/M transition 121. As FOXM1a lacks transcriptional activities, SRSF3-mediated FOXM1b and FOXM1c transactivate PLK1 and Cdc25B expression to control cell cycle progression. Interestingly, depletion of SRSF3 seems to lead to different type of cell cycle arrest in different cell types. In HCT116 colon cancer cells, SRSF3 knockdown induces G1 cell cycle arrest due to the downregulation of cyclin D1 and E2F1, which are essential for G1-to-S cell cycle progression 114. However, SRSF3 may also inhibit G1 phase progression by suppressing Rac family small GTPase 1 (Rac1) exon 3b splicing and the production of an oncogenic Rac1b, which plays an important role in G1 phase 122.

ILF3 is involved in the regulation of cell proliferation in cancer cells 123. *ILF3* gene produces multiple RNA isoforms *via* the alternative splicing of exon 19 to 21. The inclusion of *ILF3* exon 19 leads to the usage of an alternative proximal polyadenylation site. Moreover, *ILF3* exon 20 and 21 are two alternative exons. We found that SRSF3 increases exon 19 inclusion and the usage of a proximal 3' splice sit to produce oncogenic ILF3 isoform-2, as well as the inclusion of exon 20 and 21 to produce oncogenic ILF3 isoform-1, by interacting with several motifs in exon 18. In contrast, tumor suppressive ILF3 isoform-5 and -7 expression were inhibited by SRSF3 80.

MDM4 protein inactivates p53 function and is often upregulated in highly proliferating stem cells or cancer cells. The increased MDM4 protein level in these cells is due to increased translational activities as its mRNA level often remains unchanged. Mechanically, *MDM4* exon 6 is an alternative exon. Exclusion of exon 6 disrupts its open reading frame to produced an unstable short RNA isoform as a NMD target. SRSF3 promotes exon 6 inclusion and full-length MDM4 protein expression. An oligonucleotide targeting the SRSF3 binding sites in exon 6 represses exon 6 inclusion and MDM4 protein expression, and thereby, inhibits the development of melanoma and diffuse large B cell lymphoma 124.

Glioblastoma is one of the most aggressive brain tumors characterized by fast growth and spread, and chemotherapy resistance. SRSF3 is required for glioblastoma cancer cell proliferation and tumorigenesis by promoting exon 7 inclusion and protein stability of transcription factor ETV1 (ETS variant 1), which enhances cell proliferation and invasion^82^.

#### 5.2.2 SRSF3 inhibits apoptosis

Increased SRSF3 expression is necessary for indefinite growth of cancer cells and prevents cancer cell apoptosis. In contrast, depletion of SRSF3 in many types of cancer cells examined inhibits cell proliferation and induce cell apoptosis initially found in U2OS cells 12. Subsequently, a series of studies have revealed that SRSF3 knockdown induces significant high level of caspase-3 cleavage, condensed and fragmented nuclei, and cell proliferation inhibition in U2OS, SW480 (a human colon adenocarcinoma cell line) 125, and ovarian cancer cells 119. HIPK2 (Homeodomain-interacting protein kinase-2) induces apoptosis. SRSF3 regulates alternative splicing of *HIPK2* exon 8 to prevent HIPK2-related apoptosis 114, but increases the production of anti-apoptotic protein BCL2 in colon cancer cells 114.

On the other hand, SRSF3 has a role in anti-apoptosis and promotes exon 9 skipping of caspase-2 126. Caspase-2 (CASP2) exon 9 is alternative exon. Exon 9 inclusion produces pro-apoptotic caspase-2L isoform, while exon 9 exclusion produces anti-apoptotic caspase-2S isoform.

#### 5.2.3 SRSF3 protects cells from senescence

Srsf3 expression is significantly reduced in spleen and muscle of old mice compared with young mice 127. It is not surprising that SRSF3 may have an anti-aging function, because reduced SRSF3 expression leads to cellular senescence in normal human fibroblast cell line 48, human umbilical vein endothelial cells, and mouse fibroblast cell line 99. SRSF3 protects cell from senescence. In normal human fibroblasts, SRSF3 knockdown induces cellular senescence by increasing p53 (TP53) phosphorylation at serine 15, and production of p53β isoform, which can promotes p53-mediated senescence 48. Human fibroblasts undergoing replicative senescence exhibit reduced expression of SRSF3. SRSF3 knockdown also increased p53β expression and senescence in neural astrocytes 128. In principle, SRSF3 promotes the inclusion of a p53β-specific exon *via* interacting with three motifs, UUUCAAA, UACUUGAC, and UACUUCCU 48. SRSF3 prevents the usage of proximal polyadenylation sites in many genes associated with senescence pathways, such as PTEN and inhibits the expression of these genes in senescence-related phenotypes 99. Cells undergoing senescence remain viable but fail to proliferate. Therefore, senescence can suppress tumorigenesis by blocking uncontrolled cell growth in cancer cells 129. Thus, SRSF3 is an inhibitor of cellular senescence and promotes cell growth.

#### 5.2.4 SRSF3 represses autophagy

Autophagy helps cells to recycle materials and is essential in physiological and pathological conditions. SRSF3 is significantly downregulated during hypoxia-induced autophagy. Knockdown of SRSF3 induces significant cellular autophagic activity, whereas SRSF3 overexpression inhibits autophagy in oral squamous cell carcinoma cells. Mechanically, SRSF3 inhibits the expression of p65 (RELA) and FOXO1 to reduce expression of BECN1, a key regulator of autophagy 130.

#### 5.2.5 SRSF3 is required for the homologous recombination-mediated DNA repair

In ovary cancer cells, SRSF3 inhibits DNA double-strand break, and promotes homologous recombination-mediated DNA repair. This is because SRSF3 regulates the expression of the genes involved in DNA double-strand break repair. Knockdown of SRSF3 expression decreases the expression of BRCA1, BRIP1, and RAD51, which are responsible for homologous recombination-mediated DNA repair, at both transcription and protein levels, but has no effect on RNA splicing of these genes 131. In pancreatic cancer, SRSF3 increases the expression of lncRNA ANRIL long isoform by recognizing its m6A modification. The long isoform of lncRNA ANRIL promotes DNA homologous recombination repair by binding to DNA damage sites to recruit DNA repair proteins RAD50, BRCA1, BRCA2, and RAD51 107. Knockout of mouse Srsf3 in hepatocytes causes DNA damage and the decreased expression of Xrcc1 (X-ray repair cross-complementing protein 1), Msh2 (mutS homolog 2), and Xpd (xeroderma pigmentosum group D) 56.

#### 5.2.6 SRSF3 enhances cell reprogram

During the reprograming progress in mouse embryonic fibroblast, Srsf3 expression level undergoes a relative lower increase, followed by a sharp increase, which is correlated with the increases of cell proliferation genes and activation of genes of stem cell maintenance 103. Overexpression of Srsf3 in mouse embryonic fibroblast significantly enhances the reprogramming by binding *Nanog* mRNA and promoting its nuclear export and protein expression 103. Moreover, increased expression of mouse Srsf3 has been noted in induced pluripotent stem cells (iPSCs) when compared with parent somatic cells. Knockdown of mouse Srsf3 displays no effect on somatic cell proliferation, but significantly suppresses the efficiency of somatic cell reprogramming 132.

#### 5.2.7 SRSF3 regulates metabolism

PKM (Pyruvate kinase M) gene produces two isoforms, M1 and M2, *via* mutual splicing of exon 9 or 10 133. M1 promotes oxidative phosphorylation and is mainly expressed in normal muscle and brain tissues, while M2 promotes aerobic glycolysis and is mainly expressed in embryonic and cancer tissues 134. A potent SRSF3-binding exonic splicing enhancer in exon 10 is responsible for M2 expression. Knockdown of SRSF3 significantly decreases lactate production and cancer cell proliferation by decreasing exon 10 inclusion which is independently of exon 9 inclusion 135. In contrast, knockdown of SRSF3 increases M1 protein level and thus, the ratio of M1 vs M2 protein levels, to induce the energy metabolic shift from glycolysis and oxidative phosphorylation in colorectal cancer cells 116. This SRSF3/PKM/glycolytic pathway also happens in dendritic cells 136.

Natural and synthetic glucocorticoids bind GR (glucocorticoid receptor) to maintain various cellular metabolic and homeostatic functions 137. GR contains two isoforms generated by alternative splicing of exon 9 of NR3C1 gene. The usage of proximal 5' splice site of exon 9 produces GRα, which is the classic receptor for glucocorticoid receptor signaling. Whereas the usage of distal 5' splice site of exon 9 produces GRβ, which plays the dominant negative effects and is upregulated in a variety of glucocorticoid resistant diseases 137. SRSF3 promotes the usage of proximal splice site of exon 9 and the product of GRα in breast cancer cells 138 or THP-1 cells 139. However, SRSF3 in HeLa cell does not regulate GR exon 9 splicing 140, indicating that the effects of SRSF3 on GR exon 9 splicing is cell type dependent. Interestingly, SRSF3 enhances glucocorticoid receptor signaling by increasing GRα expression and is upregulated upon cortisol stimulation^138^.

### 5.2.8 SRSF3 and stress responses

Stress granules are membraneless cytoplasmic RNA particles containing stalled mRNA-protein complexes in response to various stresses 141. SRSF3 is a component of stress granules and is required for its assembly, indicating its important role in mRNA metabolism in the cytoplasm 142. Under arsenite-induced oxidative stress, SRSF3 is neddylated in Lys85 and promotes stress granule aggregation in human osteosarcoma U2OS cells 26. However, SRSF3 in hepatocytes is neddylated at Lys11 and degraded by proteasome under palmitic acid-induced oxidative stress 25. These results suggests that the distribution, function, and fate of SRSF3 in different cells are regulated possibly by the neddylation in its different Lys sites under various stress conditions.

### 5.3 SRSF3 promotes tumorigenesis (Figure 5)

#### 5.3.1 SRSF3 in malignant transformation progress

SRSF3 expression is gradually increased during the malignant transformation progress from normal oral mucosal, dysplasia (precancerous lesions), to oral squamous cell carcinoma 117. In a mouse model of mammary tumorigenesis, Srsf3 expression is dramatically up-regulated during the progress of mammary cancer in both mRNA and protein levels 143. We found that SRSF3 in overexpression could transform mouse embryonic fibroblast and induce tumors in nude mice *via* upregulating the expression of FoxM1 and its downstream targets Cdc25B and PLK1 12, as well as promoting the expression of ILF3 isoform-1 and isoform-2 80. These results suggest SRSF3 play an important role in malignant transformation progression.

#### 5.3.2 SRSF3 in cancer invasion and metastasis

SRSF3 may promote cancer invasion and metastasis *via* several mechanisms.

MAP4K4 (Mitogen-activated protein kinase kinase kinase kinase 4) regulates cell transformation and invasion 144. We found SRSF3 is required for the inclusion of MAP4K4 novel exon 23alt 16. SRSF3 also promotes the inclusion of *MAP4K4* exon 16 *via* CU rich elements to induce the expression of epithelial mesenchymal transition (EMT)-related genes 67.

CD44 is a cell surface hyaluronan-binding glycoprotein and a stem cell biomarker, which enhances cell migration by mediating cell-cell and cell-extracellular matrix interactions 145. SRSF3 increases the inclusion of exon v8-v10 of CD44 to produce oncogenic isoform CD44E in gastric cancer 146 and promotes the inclusion of variable exons of CD44 isoforms in triple-negative breast cancer (TNBC) 147. SRSF3 recognizes an exonic splicing enhancer (5'-CUUCGAUCAACGCCACGCCA-3') in CD44 exon v9 45.

SRSF3 can also promote metastasis and anoikis resistance by enhancing the inclusion of *CPEB2* exon 4 *via* binding to a “CAUCC” splicing enhancer, and producing full-length CPEB2B protein to activate the translation of TWIST1 and HIF1α in triple negative breast cancer 113. In a mouse breast cancer cell line, Srsf3 knockdown significantly reduces cellular migration, invasion *in vitro*, and lung metastasis *in vivo*. Mechanically, SRSF3 forms a complex with TDP43 and promotes the exclusion of PAR3 (partitioning defective protein 3) exon 12 to produce an oncogenic isoform in coordination with splicing factor TDP43, while full-length PAR3 is a metastasis inhibitor 95.

#### 5.3.3 SRSF3 and cancer drug resistance

Gemcitabine, a widely used chemotherapy drug, kills cancer cells by inhibiting DNA replication and cell division. However, cancer cells often develop resistance to gemcitabine 148. SRSF3 is highly expressed in gemcitabine-resistant pancreatic cancer cells. The increased expression of SRSF3 enhances gemcitabine resistance of pancreatic cancer cells both *in vitro* and *in vivo*, whereas silence of SRSF3 by siRNAs exhibits an opposite effect.

#### 5.3.4 SRSF3 promotes angiogenesis

Angiogenesis is essential for the development of cancer. Knockdown of SRSF3 expression significantly inhibits VEGF (vascular endothelial growth factor) expression in colorectal cancer cells, cancer cell migration, and tube formation by human umbilical vein endothelial cells (HUVECs) *in vitro* via a proangiogenic SRF (serum response factor) gene. SRSF3 is co-expressed with SRF in colorectal cancer tissues, and required for SRF expression 149.

### 5.4 SRSF3 is a negative regulator of immune response

#### 5.4.1 SRSF3 in inflammation and innate immune responses

SRSF3 regulates innate immune responses. SRSF3 inhibits IL-1β expression in a monoclonal cell line, THP-1 cells, upon the challenge of *Escherichia coli*. Bacterial treatment significantly reduces SRSF3 expression to allow IL-1β expression in primary monocytes 150. In dendritic cells, histone deacetylase (HDAC) inhibitor TSA upregulates SRSF3 and downregulates pro-inflammatory cytokines, including IL-1β, IL-10, and IL-12 136.

A set of innate immune associated genes, such as *Saa3*, *Ccl5*, *Ccl3*, and *Lcn2*, are highly transcribed, but not efficiently translated under inflammation stimulation in brain microglial cells. Srsf3 binds to the 3' UTR of these genes and represses their translation. Interestingly, Srsf3 expression is upregulated by LPS-activated mouse brain microglial cells and may suppress uncontrolled inflammation responses and tissue damage 151.

#### 5.4.2 SRSF3 and cancer immunotherapy

SRSF3 is involved in regulating the expression of genes related to cancer immunotherapy. PD-1 (Programmed cell death-1) is an important immunosuppressor molecule for cancer immunotherapy 152. TGF-β1 induced Smad3 to transactivate PD-1 expression in T cells to restrict their antitumor function 153. Besides Smad3, TGF-β1 also promotes PD-1 expression by two mechanisms mediated by SRSF3. TGF-β1 promotes SRSF3 expression. Upregulated SRSF3 binds to and prolongs PD-1 mRNA half-life by increasing its stability, and promotes its nuclear export by the interaction with NXF1 52.

In B-cell acute lymphoblastic leukemias (B-ALL), SRSF3 expression seems to be beneficial for patients. CD19 is a target antigen in B-ALL. Mutation or skipping of CD19 exon 2 induces the resistance to chimeric antigen receptor-armed T cell (CART-19) treatment 154. SRSF3 is required for the inclusion of exon 2 by interacting with the [A/T]CAAC[A/G] motifs, and is downregulated in relapsed leukemias after immunotherapy 154.

Roles of SRSF3 in anti-tumor immune response can be exemplified by YAP1 (Yes-associated protein 1), an oncogenic co-transactivator. YAP1-2γ contains a γ fragment encoded by an alternative exon 6 and exhibits significant anti-tumor activity by enhancing CCL2 expression and attracting massive macrophage infiltration. SRSF3 promotes exon 6 inclusion and results in the upregulation of CCL2. Oncogenic KRAS downregulates SRSF3 expression, YAP-1 exon 6 inclusion, and thus, isoform YAP1-2γ expression 155. Altogether, although SRSF3 negatively regulates inflammation and innate immune responses, SRSF3 may play double-edged roles in cancer immunotherapy.

### 5.5 Oncogenic viruses take advantage of SRSF3

Persistent infections with high-risk human papillomaviruses (HPV) lead to development of cancers. Almost all cervical cancer is caused by high-risk HPV. We discovered that SRSF3 interacted with a HPV16 A/C-rich ESE motif (nt 3488 to 3516), by which SRSF3 inhibits HPV16 later gene expression, but increases the expression of viral early genes. In cervix epithelium, SRSF3 expression gradually decreases from the undifferentiated basal layers to the highly differentiated upper layers. The viral late genes, L1 and L2, can only be expressed in the upper layers within the keratinocytes with reduced SRSF3 expression 34. Similarly, we also discovered an ESE motif (nt 3520 to 3550) in HPV18, through which SRSF3 suppressed HPV18 L1 and E2 expression 62. Importantly, SRSF3 is required for the expression of both HPV18 and HPV16 early and oncogenic genes, E6 and E7, in HPV18-positive HeLa and HPV16-positive CaSki cells, two commonly used cervical cancer cell lines 34.

Kaposi sarcoma-associated herpesvirus (KSHV) RNA-binding protein ORF57 (Mta) functions as a viral splicing factor and a key post-transcriptional regulator in viral gene expression 156 . SRSF3 inhibits KSHV K8β splicing by binding to a suboptimal K8β intron. The N-terminal ORF57 binds the SRSF3 RRM, prevents SRSF3 association with the K8β intron, and promotes K8β splicing 157. Moreover, other targets of SRSF3 were also reversely regulated by ORF57, which suggested that ORF57 might regulate cellular gene splicing *via* SRSF3 during KSHV infection 157.

SRSF3 is also used by other viruses for replication. During poliovirus infection in neuroblastoma cells, SRSF3 migrates from the nucleus to the cytoplasm, and co-localizes to poliovirus RNA with PCBP2 *via* its RS domain 158. PCBP2 binds to the major stem-loop structure (stem-loop IV) in the IRES of poliovirus RNA and facilitates the translation initiation^159^, ^160^. Poliovirus reduces cellular translation, but translates its own RNA by the IRES (internal ribosome entry sites)-mediated translation, which is enhanced by recruiting PCBP2 and SRSF3 158, 161.

In herpes simplex virus (HSV-1), viral mRNA export is mainly mediated by HSV-1 ICP27 protein. However, SRSF3 can assist the export of ICP27-mediated viral mRNA export. Depletion of SRSF3 led to about a 10-fold decrease in virus yield 162

In oncogenic Epstein-Barr virus associated with development of Burkitt lymphoma, nasopharyngeal carcinoma, and Hodgkin lymphoma, viral EB2 protein (also called SM, Mta, and BMLF1), a KSHV ORF57 homolog, interacts with the RS domain of SRSF3, and activates a cryptic alternative 5' splice site in intron 23 of STAT1 to produce a dominant negative repressor of STAT1 protein 163. STAT1 is a mediator of interferon (IFN) signal pathway. Thus, EBV EB2 protein uses SRSF3 to disrupt IFN signal pathway. SRSF3 destablizes viral intronless RNAs by interacting with nuclear RNA exosome and its adaptor complex NEXT in the absence of EBV EB2, but this effect could be counteracted by viral EB2 via interacting with SRSF3 104.

## 6. SRSF3 functions in other human diseases

### 6.1 SRSF3 and cardiac diseases

Mouse Srsf3 is essential for both heart development and function. Depletion of Srsf3 specifically in embryonic heart causes failure of cardiomyocyte proliferation, while depletion of Srsf3 in cardiomyocytes of adult mice causes severe systolic dysfunction and death 47. Srsf3 depletion causes mitochondrial matrix swelling and disorganized cristae in cardiomyocytes, accompanied by a significant reduction in complex I-driven respiration due to the decreased mitochondrial DNA and expression of oxidative phosphorylation-related genes, such as *Ndufb8*, *Sdhb*, and *Mtco1*, and aberrant alternative splicing of mitochondrial genes, such as *Bag3* and *Atg13* 164.

### 6.2 SRSF3 and neurological diseases

SRSF3 is overexpressed in Alzheimer's disease (AD) neuronal cells, which is attributed to the pathological Aβ42 fibril stimulation 165. Increased SRSF3 promotes the inclusion of alternative exon 19 of TrkB (tropomyosin receptor kinase B) receptor, which mediates the signal transduction of BDNF (brain-derived neurotrophic factor) to support neuronal survival, growth, and differentiation. The inclusion of *TrkB* alternative exon 19 leads to encoding a truncated TrkB protein lacking the tyrosine kinase domain. Truncated TrkB protein interferes the BDNF signal transduction and contributes to AD 165.

Wnt1-Cre conditional knockout of mouse Srsf3 led to facial cleft in mouse due to the defective proliferation of cranial neural crest cells and the dysregulated alternative splicing of some protein kinases in PDGFRα (platelet-derived growth factor receptor alpha) signaling pathway, such as *Limk2*, *Dmpk* and *Prkd2* genes 30.

### 6.3 SRSF3 and development

Megakaryocyte specific knockout of mouse Srsf3 blocks the maturation of megakaryocytes and the deposition of distinct megakaryocyte RNAs into platelets, leading to dramatically decreased platelet amount and dysfunctional platelets 166.

Srsf3 is essential for mouse liver development. Hepatic specific knockout of Srsf3 often results in early neonatal and perinatal death with significantly smaller body size than wild-type mice. The survived Srsf3 knockout mice also shows a significant smaller body size and liver, growth retardation, and disturbed hepatic architecture 167 because Srsf3 knockout impairs hepatocyte maturation by induction of aberrant RNA splicing of the genes involved in the regulation of glucose and lipid metabolism, such as *Hnf1α*, *Ern1*, *Hmgcs1*, *Dhcr7* and *Scap* 167. G6PD (Glucose-6-phosphate dehydrogenase) is an enzyme involved in lipogenesis in liver. Starvation induces retention of the introns 11 and 12 around exon 12 of *G6PD* to reduce G6PD expression. Conversely, nutrients stimulates splicing of the introns and to produce G6PD protein 168. There is a regulatory element in the exon 12 for its splicing 169. SRSF3 binds this element and promotes exon 12 inclusion and splicing of the introns 11 and 12, and thus, helps the cells to produce more G6PD protein upon nutrient 54. HnRNP K, hnRNP L, and hnRNP A2/B1 bind the element and suppress exon 12 inclusion 170, 171.

## 7. Current progress in developing chemical inhibitors to control SRSF3 expression

SRSF3 overexpression is partially resulted from the reduced inclusion of its poison exon 4 and the impaired autoregulation in cancers 21. An antisense oligonucleotide blocking an exonic splicing suppressor in the exon 4 can significantly increase inclusion of the exon 4, leading to inhibit SRSF3 expression and suppress proliferation of oral squamous cell carcinoma 172 and breast cancer cells 173.

Recently, a novel SRSF3 inhibitor, namely SFI003, has been developed. By screening compounds from commercial databases, SFI003, among dozens of its derivatives, has been found to be the most potent inhibitor on SRSF3 activities, with a lowest IC_50_ value, in colorectal cancer cells. Moreover, SFI003 induces the neddylation-dependent degradation of SRSF3 protein and promotes apoptosis, thereby inhibiting cell proliferation, migration, and tumor formation 174.

Other chemicals also have the abilities to reduce SRSF3 expression. Amiloride, a diuretic, induces hypo-phosphorylation and downregulation of SRSF3 and SRSF1, leading to alteration of RNA splicing patterns in many genes and cell cycle disruption in hepatocellular carcinoma cells 175.

Digitoxin, a structural analogue of digoxin, also regulates a large set of alternative RNA splicing events by repressing SRSF3 expression 176. Mechanically, digoxin decreases SRSF3 expression by inducing its protein ubiquitination and degradation. Conversely, DARPP-32 (dopamine and cAMP-regulated phosphoprotein of molecular weight 32 kDa), a key signaling mediator in several neurotransmitter pathways 177, encoded by PPP1R1B gene, binds SRSF3 protein and decreases digitoxin-induced ubiquitination of SRSF3 protein, and thus significantly increases SRSF3 stability 146.

## 8. Summary

SRSF3 protein and its alternative exon 4 are well conserved during evolution from animal kingdom. As a multifunctional RNA-binding protein and splicing factor, auto- and balanced regulatory mechanisms in SRSF3 expression in host cells are essential in maintaining the proper expression level of SRSF3 protein, which is often disrupted in cancers. SRSF3 is involved in many cellular processes by regulating almost all aspects of RNA biogenesis and processing of many target genes. Thus, any disturbance of SRSF3 expression and modification will contribute to turbulence of normal cell functions and the pathological outcomes. As a proto-oncogene, overexpression of SRSF3 has proven to induce cell transformation and tumor formation and maintenance. Conceivably, all types of cancers examined to date exhibit increased expression of SRSF3, which is essential for keeping cancer cell proliferation and cell cycle progression. Deficiency of SRSF3 also causes severe diseases by deregulation of RNA processing of many target genes. Although restoring a proper expression level of SRSF3 in specific cells may be the key for curing SRSF3 deficiency-induced diseases, blocking SRSF3 expression and its functions in cancer cells by small molecules will be an exciting and promising roadmap for future cancer therapies.

## Supplementary Material

Supplementary figures.

## Figures and Tables

**Figure 4:**
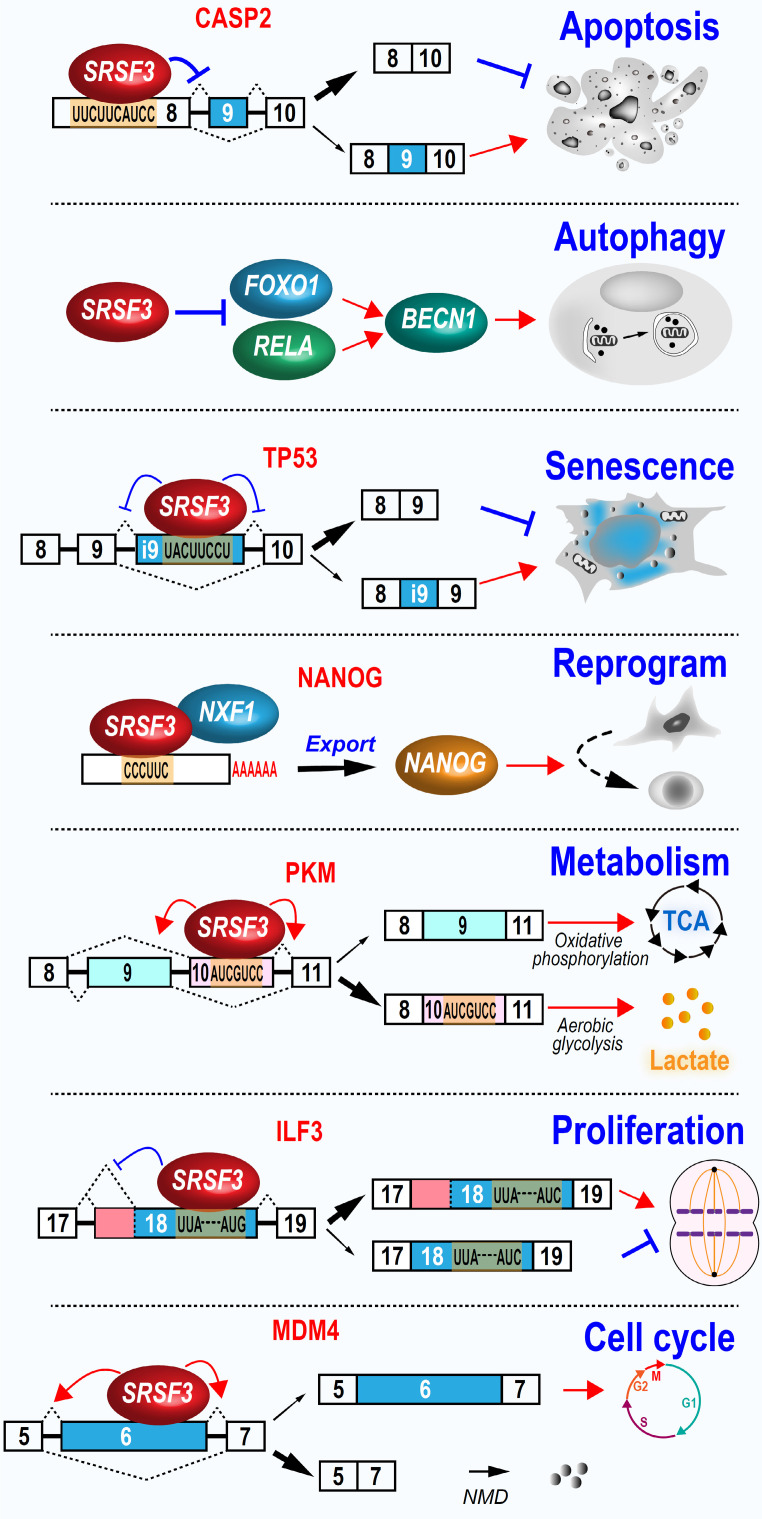
Cellular functions of SRSF3. SRSF3 regulates wide-range of cellular functions associated with tumorigenesis, including cell proliferation, cell cycle progression, cell apoptosis, autophagy, senescence, reprogram, and metabolism.

**Figure 5:**
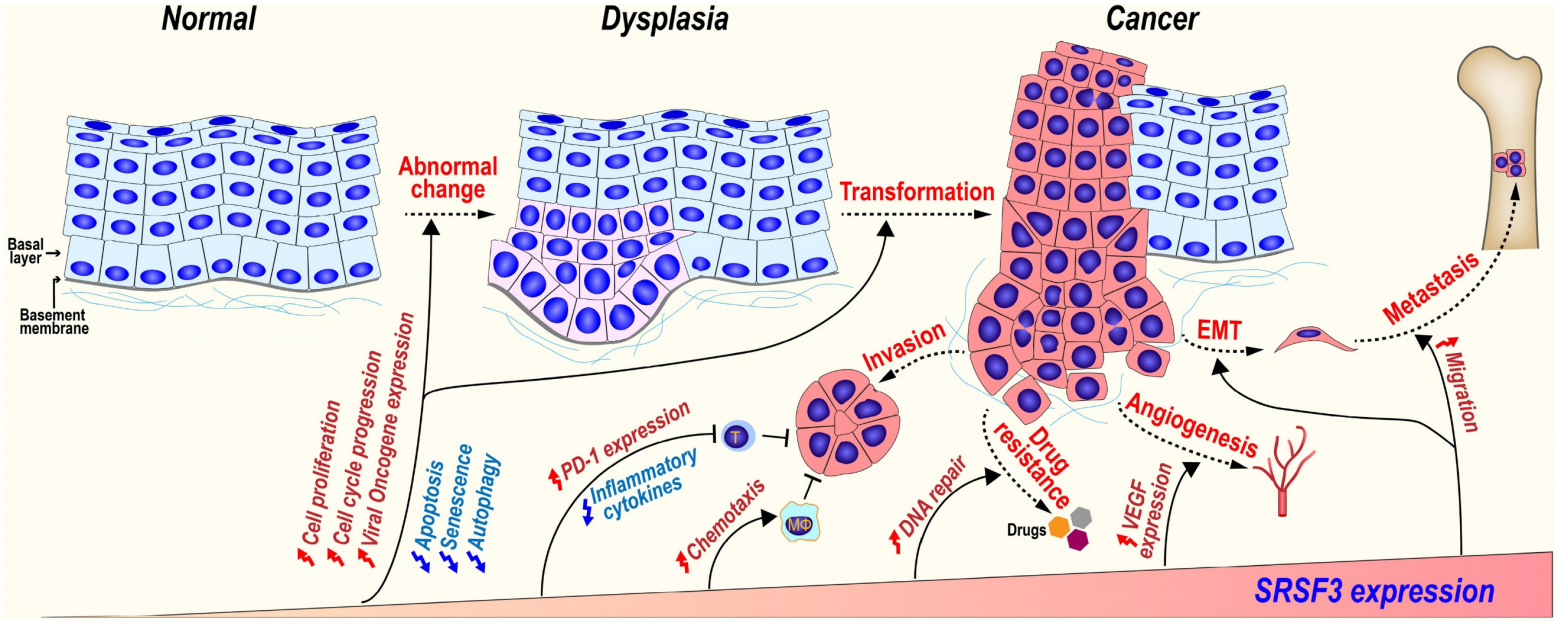
SRSF3 plays important roles during tumorigenesis. SRSF3 is overexpressed in dysplasia and cancer tissues. SRSF3 in overexpression promotes cell malignant transformation and the invasion, metastasis, angiogenesis, drug resistance, and immunosuppression of cancers.

**Table 1:** Targets and/or binding motifs of SRSF3

Gene or viral RNA	Exon or intron	Binding motif sequence	Function of SRSF3	Pathways	Ref.
BCL-2 interacting cell death suppressor (BIS)	Exon 3	UCAUCCUCAUC	Enhancing exon inclusion	Apoptosis	178
BPV-1	Late RNA exon 2	CACCACCACC	Enhancing exon 2 inclusion	BPV-1 L1	34
Calcitonin/calcitonin gene-related peptide	Intron 4	CUCCGCUCCUCUUCCAGGUAAGAC	Enhancing exon inclusion	CGRP (Calcitonin gene-related peptide)	6
CASP2	Exon 8	UCUUCAUC	Enhancing exon skipping	Apoptosis	126
CD44	Exon v9	CUUCGAUCAACGCCACGCCA	Enhancing exon inclusion	CD44	45
CHK1	Exon 3	/	Enhancing exon inclusion	Cell cycle control	16
CKLF	Exon 3	/	Enhancing exon inclusion	Inflammation	16
CLINT1	Exon 11	/	Enhancing exon inclusion	Vesicle-mediated transport	16
CPEB2	Exon 4	CAUCC	Enhancing exon inclusion	Epithelial to mesenchymal transition (EMT)	113
DDX5	Exon 12	/	Enhancing exon inclusion	β-catenin	16
EP300	Exon 14	CCAGCC	Enhancing exon inclusion	Chromatin remodeling	16
FOXM1	Exon 9	/	Enhancing exon skipping	Cell cycle progression	12
G6PD	Exon 12	AGCCCCAGCC	Enhancing exon inclusion	Insulin	54
HER2	Exon 15	UGAUG, AGATG	Promoting upstream or downstream exon skipping)	HER2 signaling	84
HPV16 early RNA	Exon 2	CACCGGAAACCCCUGCCACACCAC	Enhancing exon 2 inclusion	HPV16 E6/E7	34
HPV18 early RNA	Exon 2	ACCGCA, CCAGAC	Enhancing exon 2 inclusion	HPV18 E6/E7	62
H2al1k	Exon 1	ACAACAAGAAGACGCGCAUCAU	RNA export	Chromatin assembly	10
ILF3	Exon 18	AUCAACUUUUUACUCCAAUUUCCUCCA and UUAACUUCUCCCGCCUCUUGUAAUG	Enhancing exon 18 inclusion	Serine-Glycine-One-Carbon (SGOC) pathway	80
INSR	Exon 11	CUCUUC	Enhancing exon inclusion	Insulin	92
KIF23	Exon 18	/	Enhancing exon inclusion	β-catenin	16
MAP4	Exon 10	/	Enhancing exon inclusion	Cell cycle progression	16
MAP4K4	Exon 16	GUCUUUUUCCA	Enhancing exon inclusion	RBM4/SRSF3/MAP4K4	67
MBNL1	Exons 5 and 7	CAACC, CACCCGC	Enhancing exon inclusion	Expanded dsCUG binding and RNA splicing	94
MELK	Exon 11	/	Enhancing exon inclusion	Cell cycle progression	16
NANOG	Exon 1	CCCUUC, GCAUCG, CAUC	Nuclear export	Reprogram	103
PABPC1	Exon 10 and 11	/	Enhancing exon inclusion	mRNA metabolism	16
PKM	Exon 10	AUCGUCC	Enhancing exon inclusion	Energy metabolism	135
PKP4	Exon 7	/	Enhancing exon inclusion	Rho GTPases pathway	16
PUS3	Exon 3	/	Enhancing exon inclusion	tRNA processing	16
SMC2	Exon 3 and 4	/	Enhancing exon inclusion	Cell cycle progression	16
SRSF1	Exon 4	/	Enhancing exon inclusion	Splicing factor	16
SYNGAP1	Exon 14	CCUCAAC	Enhancing exon skipping	Ras-Raf-MEK-ERK pathway	95
TP53	Novel exon	UUUCAAA, UACUUGAC, and UACUUCCU	Enhancing exon skipping	TP53	48
	*In vitro* screening	CUCKUCY	Exonic splicing enhancer		88
	*In vivo* iCLIP	UCAUC, CUUCA, CAUCA, CAUCU, and UCAAC,	Binding motifs		87

/, unknown; K, guanosine or uridine; Y, cytidine or uridine.
