# Supplementary material for: Oncogenic SRSF3 in health and diseases
Source: Int J Biol Sci. 2023 Jun 12;19(10):3057–76. doi: 10.7150/ijbs.83368 (PMC10321290; doi:10.7150/ijbs.83368)
Supplement: Supplementary file 1 — Supplementary figures. [file ijbsv19p3057s1.pdf]

Figure S1. Alignment of SRSF3 exon 4 sequences of different species by using NCBI Multiple Sequence Alignment Viewer

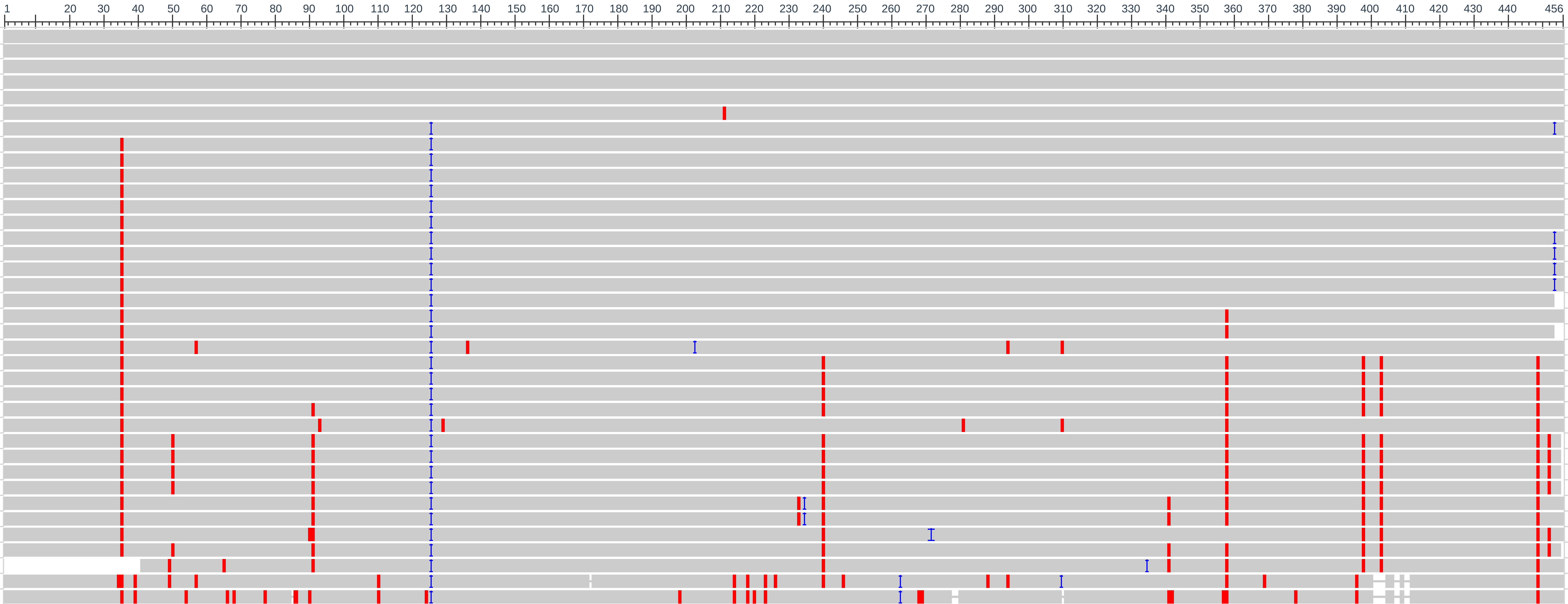

Sequence ID		Start	Alignment	End	Organism	Identity	Mismatches
Query_59323	(+)	1		456		100.00	0
XR_001718382.2	(+)	1,163		1,618	Pan troglodytes	100.00	0
XR_001503726.1	(+)	510		965	Marmota marmota marm...	100.00	0
NR_036610.2	(+)	464		919	Homo sapiens	100.00	0
NR_036613.1	(+)	471		926	Mus musculus	100.00	0
XR_004608809.1	(+)	508		963	Arvicanthis niloticus	99.78	1
XR_001297835.2	(+)	472		929	Sus scrofa	99.56	0
XR_004343625.1	(+)	481		937	Lynx canadensis	99.56	1
XR_004076600.1	(+)	488		944	Leptonychotes weddellii	99.56	1
XR_003621478.1	(+)	526		982	Eptesicus fuscus	99.56	1
XR_003611887.1	(+)	474		930	Eumetopias jubatus	99.56	1
XR_001604051.1	(+)	512		968	Miniopterus natalensis	99.56	1
XR_001597846.2	(+)	480		936	Rousettus aegyptiacus	99.56	1
XR_003508318.1	(+)	500		957	Bos indicus x Bos taurus	99.34	1
XR_002310364.1	(+)	493		950	Odocoileus virginianus t...	99.34	1
XR_826010.1	(+)	500		957	Bison bison bison	99.34	1
XR_005021819.1	(+)	463		920	Balaenoptera musculus	99.34	1
XM_015560868.1	(+)	501		954	Myotis davidii	99.56	1
XR_005010857.1	(+)	465		921	Trichosurus vulpecula	99.34	2
XM_007483785.2	(+)	562		1,015	Monodelphis domestica	99.34	2
XR_006680519.1	(+)	52		509	Echinops telfairi	98.47	5
XR_001292554.1	(+)	452		908	Apteryx mantelli mantelli	98.47	6
XR_775443.1	(+)	165		621	Haliaeetus leucocephalus	98.47	6
XR_718559.1	(+)	167		623	Haliaeetus albicilla	98.47	6
XR_728303.1	(+)	1,265		1,721	Tinamus guttatus	98.25	7
XR_005451838.1	(+)	286		742	Tachyglossus aculeatus	98.25	7
XR_001991825.2	(+)	458		913	Serinus canaria	97.81	9
XR_003916067.1	(+)	345		800	Manacus vitellinus	97.81	9
XR_001402830.1	(+)	455		910	Sturnus vulgaris	97.81	9
XR_004981636.1	(+)	450		905	Molothrus ater	97.81	9
XR_004550334.1	(+)	451		908	Melopsittacus undulatus	97.60	9
XR_003989636.1	(+)	453		910	Strigops habroptila	97.60	9
XR_624727.1	(+)	116		574	Acanthisitta chloris	97.60	8
XR_005562939.1	(+)	451		906	Pipra filicauda	97.59	10
XM_030465436.1	(+)	1		418	Calypte anna	97.37	9
XR_003944067.1	(+)	381		830	Microcaecilia unicolor	93.46	18
XR_001354548.1	(+)	459		904	Latimeria chalumnae	91.92	23

Figure S2. Alignment of SRSF3 protein sequences of different species by using online service of Clustal Omega form EMBL's European Bioinformatics Institute.

Class	Species	Protein sequence ID	Protein sequences	
	Homo sapiens (human)	NP_003008.1	MHRDSCPLDCKVYVGNLGNNGNKTELERAFGYYGPLRSVWVARNPPGFAFVEFEDPRDAA	60
	Pan troglodytes (chimpanzee)	XP_001173216.1	MHRDSCPLDCKVYVGNLGNNGNKTELERAFGYYGPLRSVWVARNPPGFAFVEFEDPRDAA	60
	Macaca mulatta (Monkey)	XP_014991738.1	MHRDSCPLDCKVYVGNLGNNGNKTELERAFGYYGPLRSVWVARNPPGFAFVEFEDPRDAA	60
	Canis lupus familiaris (dog)	XP_038539266.1	MHRDSCPLDCKVYVGNLGNNGNKTELERAFGYYGPLRSVWVARNPPGFAFVEFEDPRDAA	60
	Sus scrofa (pig)	XP_020954305.1	MHRDSCPLDCKVYVGNLGNNGNKTELERAFGYYGPLRSVWVARNPPGFAFVEFEDPRDAA	60
	Vulpes lagopus (fox)	XP_041618874.1	MHRDSCPLDCKVYVGNLGNNGNKTELERAFGYYGPLRSVWVARNPPGFAFVEFEDPRDAA	60
Mammalia	Ovis aries (sheep)	XP_011955800.1	MHRDSCPLDCKVYVGNLGNNGNKTELERAFGYYGPLRSVWVARNPPGFAFVEFEDPRDAA	60
	Equus caballus (horse)	XP_001499880.1	MHRDSCPLDCKVYVGNLGNNGNKTELERAFGYYGPLRSVWVARNPPGFAFVEFEDPRDAA	60
	Felis catus (cat)	XP_003986117.1	MHRDSCPLDCKVYVGNLGNNGNKTELERAFGYYGPLRSVWVARNPPGFAFVEFEDPRDAA	60
	Mus musculus (mouse)	NP_038691.1	MHRDSCPLDCKVYVGNLGNNGNKTELERAFGYYGPLRSVWVARNPPGFAFVEFEDPRDAA	60
	Cricetulus griseus (hamster)	XP_003504193.1	MHRDSCPLDCKVYVGNLGNNGNKTELEHAFGYYGPLRSVWVARNPPGFAFVEFEDPRDAA	60
	Rattus norvegicus (rat)	NP_001041372.1	MHRDSCPLDCKVYVGNLGNNGNKTELERAFGYYGPLRSVWVARNPPGFAFVEFEDPRDAA	60
	Castor canadensis (beaver)	XP_020035159.1	MHRDSCPLDCKVYVGNLGNNGNKTELERAFGYYGPLRSVWVARNPPGFAFVEFEDPRDAA	60
	Balaenoptera musculus (blue whale)	XP_036724491.1	MHRDSCPLDCKVYVGNLGNNGNKTELERAFGYYGPLRSVWVARNPPGFAFVEFEDPRDAA	60
	Passer montanus (sparrow)	XP_039555293.1	MHRDSCPLDCKVYVGNLGNNGNKTELERAFGYYGPLRSVWVARNPPGFAFVEFEDPRDAA	60
Aves	Tyto alba (barn owl)	XP_032844819.1	MHRDSCPLDCKVYVGNLGNNGNKTELERAFGYYGPLRSVWVARNPPGFAFVEFEDPRDAA	60
	Gallus gallus (chicken)	NP_001182483.1	MHRDSCPLDCKVYVGNLGNNGNKTELERAFGYYGPLRSVWVARNPPGFAFVEFEDPRDAA	60
	Cygnus olor (swan)	XP_040392143.1	MHRDSCPLDCKVYVGNLGNNGNKTELERAFGYYGPLRSVWVARNPPGFAFVEFEDPRDAA	60
	Varanus komodoensis (Komodo Dragon)	XP_044299976.1	MHRDSCPLDCKVYVGNLGNNGNKTELERAFGYYGPLRSVWVARNPPGFAFVEFEDPRDAA	60
Reptilia	Mauremys reevesii (tortoise)	XP_039393834.1	MHRDSCPLDCKVYVGNLGNNGNKTELERAFGYYGPLRSVWVARNPPGFAFVEFEDPRDAA	60
	Python bivittatus (python)	XP_025019020.1	MHRDSCPLDCKVYVGNLGNNGNKTELERAFGYYGPLRSVWVARNPPGFAFVEFEDPRDAA	60
Amphibia	Microcaecilia unicolor (caecilian)	XP_030077880.1	MHRDSCPLDCKVYVGNLGNNGNKTELERAFGYYGPLRSVWVARNPPGFAFVEFEDPRDAA	60
	Bufo gargarizans (bufonid)	XP_044139482.1	MHRDSCPLDCKVYVGNLGNNGNKTELERAFGYYGPLRSVWVARNPPGFAFVEFEDPRDAA	60
			***************************:********************************	

	Homo sapiens	NP_003008.1	DAVRELDGRTLCGCRVRVELSNGEKRSRNRGPPPSWGRRPRDDYRRRSPPPRRRSPRRRS	120
	Pan troglodytes	XP_001173216.1	DAVRELDGRTLCGCRVRVELSNGEKRSRNRGPPPSWGRRPRDDYRRRSPPPRRRSPRRRS	120
	Macaca mulatta	XP_014991738.1	DAVRELDGRTLCGCRVRVELSNGEKRSRNRGPPPSWGRRPRDDYRRRSPPPRRRSPRRRS	120
	Canis lupus familiaris	XP_038539266.1	DAVRELDGRTLCGCRVRVELSNGEKRSRNRGPPPSWGRRPRDDYRRRSPPPRRRSPRRRS	120
	Sus scrofa	XP_020954305.1	DAVRELDGRTLCGCRVRVELSNGEKRSRNRGPPPSWGRRPRDDYRRRSPPPRRRSPRRRS	120
	Vulpes lagopus	XP_041618874.1	DAVRELDGRTLCGCRVRVELSNGEKRSRNRGPPPSWGRRPRDDYRRRSPPPRRRSPRRRS	120
Mammalia	Ovis aries	XP_011955800.1	DAVRELDGRTLCGCRVRVELSNGEKRSRNRGPPPSWGRRPRDDYRRRSPPPRRRSPRRRS	120
	Equus caballus	XP_001499880.1	DAVRELDGRTLCGCRVRVELSNGEKRSRNRGPPPSWGRRPRDDYRRRSPPPRRRSPRRRS	120
	Felis catus	XP_003986117.1	DAVRELDGRTLCGCRVRVELSNGEKRSRNRGPPPSWGRRPRDDYRRRSPPPRRRSPRRRS	120
	Mus musculus	NP_038691.1	DAVRELDGRTLCGCRVRVELSNGEKRSRNRGPPPSWGRRPRDDYRRRSPPPRRRSPRRRS	120
	Cricetulus griseus	XP_003504193.1	DAVRELDGRTLCGCRVRVELSNGEKRSRNRGPPPSWGRRPRDDYRRRSPPPRRRSPRRRS	120
	Rattus norvegicus	NP_001041372.1	DAVRELDGRTLCGCRVRVELSNGEKRSRNRGPPPSWGRRPRDDYRRRSPPPRRRSPRRRS	120
	Castor canadensis	XP_020035159.1	DAVRELDGRTLCGCRVRVELSNGEKRSRNRGPPPSWGRRPRDDYRRRSPPPRRRSPRRRS	120
	Balaenoptera musculus	XP_036724491.1	DAVRELDGRTLCGCRVRVELSNGEKRSRNRGPPPSWGRRPRDDYRRRSPPPRRRSPRRRS	120
	Passer montanus	XP_039555293.1	DAVRELDGRTLCGCRVRVELSNGEKRSRNRGPPPSWGRRPRDDYRRRSPPPRRRSPRRRS	120
Aves	Tyto alba	XP_032844819.1	DAVRELDGRTLCGCRVRVELSNGEKRSRNRGPPPSWGRRPRDDYRRRSPPPRRRSPRRRS	120
	Gallus gallus	NP_001182483.1	DAVRELDGRTLCGCRVRVELSNGEKRSRNRGPPPSWGRRPRDDYRRRSPPPRRRSPRRRS	120
	Cygnus olor	XP_040392143.1	DAVRELDGRTLCGCRVRVELSNGEKRSRNRGPPPSWGRRPRDDYRRRSPPPRRRSPRRRS	120
	Varanus komodoensis	XP_044299976.1	DAVRELDGRTLCGCRVRVELSNGEKRSRNRGPPPSWSRRPRDDYRRRSPPPRRRSPRRRS	120
Reptilia	Mauremys reevesii	XP_039393834.1	DAVRELDGRTLCGCRVRVELSNGEKRSRNRGPPPSWGRRPRDDYRRRSPPPRRRSPRRRS	120
	Python bivittatus	XP_025019020.1	DAVRELDGRTLCGCRVRVELSNGEKRSRNRGPPPSWGRRPRDDYRRRSPPPRRRSPRRRS	120
Amphibia	Microcaecilia unicolor	XP_030077880.1	DAVRELDGRTLCGCRVRVELSNGEKRSRNRGPPPSWGRRPRDDYRRRSPPPRRRSPRRRS	120
	Bufo gargarizans	XP_044139482.1	DAVRELDGRTLCGCRVRVELSNGEKRSRNRGPPPSWNRRPRDDYRRRSPPPRRRSPRRRS	120
			************************************.***********************	

	Homo sapiens	NP_003008.1	FSRSRSRSLSRDRRRERSLSRERNHKPSRSFSRSRSRSRSNERK	164
	Pan troglodytes	XP_001173216.1	FSRSRSRSLSRDRRRERSLSRERNHKPSRSFSRSRSRSRSNERK	164
	Macaca mulatta	XP_014991738.1	FSRSRSRSLSRDRRRERSLSRERNHKPSRSFSRSRSRSRSNERK	164
	Canis lupus familiaris	XP_038539266.1	FSRSRSRSLSRDRRRERSLSRERNHKPSRSFSRSRSRSRSNERK	164
	Sus scrofa	XP_020954305.1	FSRSRSRSLSRDRRRERSLSRERNHKPSRSFSRSRSRSRSNERK	164
	Vulpes lagopus	XP_041618874.1	FSRSRSRSLSRDRRRERSLSRERNHKPSRSFSRSRSRSRSNERK	164
Mammalia	Ovis aries	XP_011955800.1	FSRSRSRSLSRDRRRERSLSRERNHKPSRSFSRSRSRSRSNERK	164
	Equus caballus	XP_001499880.1	FSRSRSRSLSRDRRRERSLSRERNHKPSRSFSRSRSRSRSNERK	164
	Felis catus	XP_003986117.1	FSRSRSRSLSRDRRRERSLSRERNHKPSRSFSRSRSRSRSNERK	164
	Mus musculus	NP_038691.1	FSRSRSRSLSRDRRRERSLSRERNHKPSRSFSRSRSRSRSNERK	164
	Cricetulus griseus	XP_003504193.1	FSRSRSRSLSRDRRRERSLSRERNHKPSRSFSRSRSRSRSNERK	164
	Rattus norvegicus	NP_001041372.1	FSRSRSRSLSRDRRRERSLSRERNHKPSRSFSRSRSRSRSNERK	164
	Castor canadensis	XP_020035159.1	FSRSRSRSLSRDRRRERSLSRERNHKPSRSFSRSRSRSRSNERK	164
	Balaenoptera musculus	XP_036724491.1	FSRSRSRSLSRDRRRERSLSRERNHKPSRSFSRSRSRSRSNERK	164
	Passer montanus	XP_039555293.1	FSRSRSRSLSRDRRRERSLSRERNHKPSRSFSRSRSRSRSNERK	164
Aves	Tyto alba	XP_032844819.1	FSRSRSRSLSRDRRRERSLSRERNHKPSRSFSRSRSRSRSNERK	164
	Gallus gallus	NP_001182483.1	FSRSRSRSLSRDRRRERSLSRERNHKPSRSFSRSRSRSRSNERK	164
	Cygnus olor	XP_040392143.1	FSRSRSRSLSRDRRRERSLSRERNHKPSRSFSRSRSRSRSNERK	164
	Varanus komodoensis	XP_044299976.1	FSRSRSRSLSRDRRRERSLSRERNHKPSRSFSRSRSRSRSNDRK	164
Reptilia	Mauremys reevesii	XP_039393834.1	FSRSRSRSLSRDRRRERSLSRERNHKPSRSFSRSRSRSRSNERK	164
	Python bivittatus	XP_025019020.1	FSRSRSRSLSRDRRRERSLSRERNHKPSRSFSRSRSRSRSNDRK	164
Amphibia	Microcaecilia unicolor	XP_030077880.1	FSRSRSRSLSRDRRRERSLSRERNHKTSRSFSRSRSRSRSNERK	164
	Bufo gargarizans	XP_044139482.1	FSRSRSRSLSRERRRERSLSRDRNHKPSRSFSRSRSRSRSNERK	164
			***********:*********:**** **************:**	

Figure S3. Alignment of RRM domains of SR proteins by using Clustal Omega. (A) Alignment of RRM domains of SR proteins. (B) Alignment of RRM domains of SRSF3 and SRSF7.

A

```
SR protein        Sequences of RRM domain of SR proteins

SRSF11      ------VIQVTNVSPSASSEQMRTLFGFLGKIDELRLFPPDDSPLPVSSRVCFVKFHDPD   54
SRSF10      LRPPNTSLFVRNVADDTRSEDLRREFGRYGPIVDVYVPL--DFYTRRPRGFAYVQFEDVR   58
SRSF12      -----TSLFIRNVADATRPEDLRREFGRYGPIVDVYIPL--DFYTRRPRGFAYVQFEDVR   53
SRSF2       -------LKVDNLTYRTSPDTLRRVFEKYGRVGDVYIPR--DRYTKESRGFAFVRFHDKR   51
SRSF8       -------LKVDNLTYRTSPDSLRRVFEKYGRVGDVYIPR--EPHTKAPRGFAFVRFHDRR   51
SRSF3       -----CKVYVGNLGNNGNKTELERAFGYYGPLRSVWVAR-------NPPGFAFVEFEDPR   48
SRSF7       ------KVYVGNLGTGAGKGELERAFSYYGPLRTVWIAR-------NPPGFAFVEFEDPR   47
SRSF1       -GNNDCRIYVGNLPPDIRTKDIEDVFYKYGAIRDIDLKN--R---RGGPPFAFVEFEDPR   54
SRSF9       ------RIYVGNLPTDVREKDLEDLFYKYGRIREIELKN--R---HGLVPFAFVRFEDPR   49
SRSF5       ------RVFIGRLNPAAREKDVERFFKGYGRIRDIDLKR--G--------FGFVEFEDPR   44
SRSF4       ------RVYIGRLSYQARERDVERFFKGYGKILEVDLKN--G--------YGFVEFDDLR   44
SRSF6       ----MPRVYIGRLSYNVREKDIQRFFSGYGRLLEVDLKN--G--------YGFVEFEDSR   46
                   : : .:        :.  *   * :  : :               :*.*.*

SRSF11      SAVVAQHLTNTVFVDRALIVVPYAEG-----------   80
SRSF10      DAEDALHNLDRKWICGRQIEIQFAQGDRKTPNQMKAK   95
SRSF12      DAEDALYNLNRKWVCGRQIEIQFAQGDRKTP------   84
SRSF2       DAEDAMDAMDGAVLDGRELRVQ---------------   73
SRSF8       DAQDAEAAMDGAELDGRELRVQ---------------   73
SRSF3       DAADAVRELDGRTLCGCRVRVELSNG-----------   74
SRSF7       DAEDAVRGLDGKVICGSRVRVELSTGMPRR-------   77
SRSF1       DAEDAVYGRDGYDYDGYRLRVEFPR------------   79
SRSF9       DAEDAIYGRNGYDYGQCRLRVEF--------------   72
SRSF5       DADDAVYELDGKELCSERVTIEHARA-----------   70
SRSF4       DADDAVYELNGKDLCGERVIVEHARG-----------   70
SRSF6       DADDAVYELNGKELCGERVIVEHARG-----------   72
            .*  *    :        : :
```

B

```
SRSF3       CKVYVGNLGNNGNKTELERAFGYYGPLRSVWVARNPPGFAFVEFEDPRDAADAVRELDGR 60
SRSF7       -KVYVGNLGTGAGKGELERAFSYYGPLRTVWIARNPPGFAFVEFEDPRDAEDAVRGLDGK 59
             ********....* ******.******:**:****************** **** ***:

SRSF3       TLCGCRVRVELSNG----   74
SRSF7       VICGSRVRVELSTGMPRR   77
            .:**.*******.*
```
